# Mass Biosynthesis of Coumestrol Derivatives and Their Isomers *via* Soybean Adventitious Root Cultivation in Bioreactors

**DOI:** 10.3389/fpls.2022.923163

**Published:** 2022-06-21

**Authors:** Eun Jung Lee, Myoung Chong Song, Chan-Su Rha

**Affiliations:** ^1^Research and Innovation Center, AMOREPACIFIC, Yongin, South Korea; ^2^Natural Products Research Institute, College of Pharmacy, Seoul National University, Seoul, South Korea

**Keywords:** abiotic stress, artificial soil, coumestrol derivative, Glycine max, phytoalexin, plant cell culture, soybean seedling, specialized metabolism in legume crop

## Abstract

Coumestrol (CMS) derivatives are unique compounds, which function as phytoalexins; they are derived from soybean roots, following abiotic and biotic stresses. As a phytoalexin, CMS forms a defense system that enables plants to maintain their viability. However, it is still challenging to achieve the mass production of phytoalexins, which exhibit pharmacological values, *via* plant breeding. Here, the synthesis of CMS derivatives from the seedling, plant, and adventitious root (AR) of *Glycine max* were investigated under artificial light, as well as *via* a chemical elicitor treatment. In the presence of constant light, as well as under treatment with methyl jasmonate, the CMS monoglucoside (coumestrin; CMSN) and malonyl CMSN (M-CMSN) contents of the AR culture (4 weeks) increased drastically. The two CMS derivatives, CMSN and M-CMSN, were obtained as a mixture of isomers, which were identified *via* nuclear magnetic resonance analysis. These derivatives were also observed in a soybean plant that was grown on artificial soil (AS; 5 weeks) and a Petri dish (9 days) although in considerably lesser quantities than those observed in the AR culture. Compared with the two other media (AS and the Petri dish), the AR culture achieved the superior synthesis of CMSN and M-CMSN within a relatively short cultivation period (<1 month) in laboratory-scale (3 L) and pilot-scale (1,000 L) bioreactors. The isoflavone content of AR under the constant light conditions was three-fold that under dark conditions. Significant quantities of malonyl daidzin and malonyl genistin were produced in the root of AS and the seedling of Petri dish, respectively. Flavonol glycosides were not produced in the AR culture under the dark and light conditions, as well as in AS under the dark condition. However, significant contents of kaempferol glycosides were produced in the leaves of AS and seedling of Petri dish, following the light treatment. Thus, we proposed that the established soybean AR-cultivation approach represented a better method for biosynthesizing phytoalexins, such as the CMS derivatives, as plant-derived functional materials.

## Introduction

Plants exert selective effects on bacteria in bulk soil to acquire various features for metabolic fitness (Ling et al., 2022). Important microbial interactions occur among thousands of commensals, pathogens, and symbionts and plant in the rhizosphere, and these result in numerous challenges to the plant (Ling et al., 2022). Fabaceae plants exhibit distinctive symbiotic assimilation in root nodules, thereby inducing the corresponding genes in the rhizobacterial community (Dakora, 2000). Many Fabaceae plants facilitate the synthesis of signature polyphenols, such as isoflavonoids, *via* well-known biological pathways (Sugiyama et al., 2017); isoflavone conjugates undergo different modifications when plants are subjected to different stresses (biotic and abiotic) (Ahmad et al., 2017). Under certain extrinsic stresses, such as biotic and abiotic stresses, phytoalexins are converted using existing isoflavonoids *via* induced metabolic pathways (Yoshikawa, 1978). Pterocarpans are representative inducible phytoalexins that are formed through the ring closure of isoflavonoids (Uchida et al., 2017). Among the pterocarpans available in soybean, glyceollins and their derivatives have attracted enormous attention because of their biological effects (Nwachukwu et al., 2013; Bamji and Corbitt, 2017), and their biosynthetic pathways have been elucidated (Sukumaran et al., 2018; Vadivel et al., 2019).

**Figure 1:**
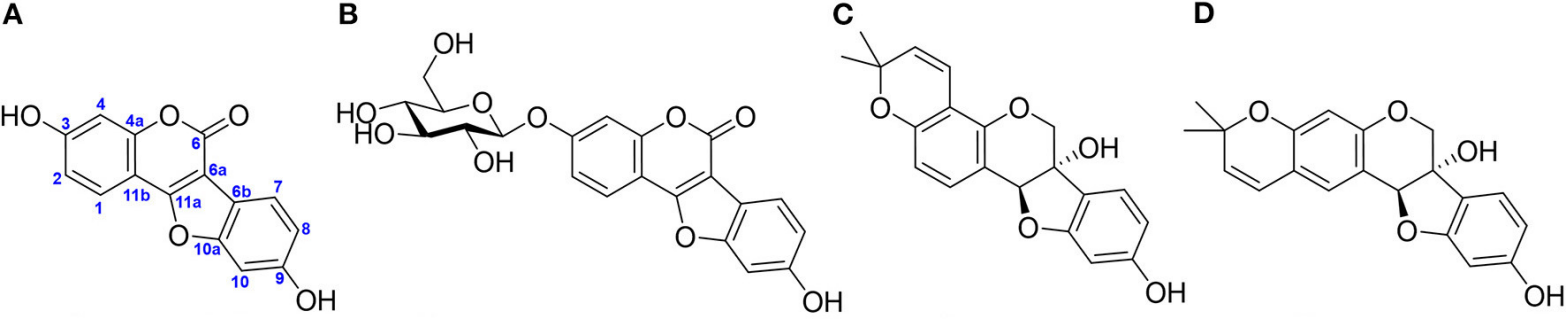
Representative phytoalexins in soybean reported in the literature. **(A)** Coumestrol (CMS), **(B)** CMS 3-*O*-glucoside, **(C)** glyceollin I, and **(D)** glyceollin II.

Moreover, compared with the large quantities of glyceollins produced in the seeds and leaves of soybean, low quantities of coumestan, the oxidation product of pterocarpans, have also been reported (Figure 1). Although coumestan derivatives were first identified in soybean roots 40 years ago (Le-Van, 1984), their studies only began a decade ago (Yuk et al., 2011a; Jeon et al., 2012; Yun et al., 2020, 2021; Cox et al., 2021; Mun et al., 2021). Considering that plant-derived coumestan exhibits a wide range of pharmacological activities (Tu et al., 2021), researchers have attempted to mass-produce coumestrol (CMS), a coumestan exhibiting phytoestrogenic activity, from soybean adventitious root (AR) (Lee et al., 2020). Although most plants produce conjugated forms of these phytochemicals (coumestan), the knowledge and applications of coumestans are only based on aglycones. Based on the biosynthetic pathway of CMS (Ha et al., 2019), we believed that conjugated or complex coumestan derivatives could be produced employing intrinsic enzymes. Further, we observed that the laboratory (lab)-scale mass production of another phytoalexin from a soybean AR culture could be achieved. Thus, here, we identified new CMS derivatives, which were biosynthesized from soybean AR, following abiotic stress. Furthermore, the soybean AR was cultivated on a pilot scale to achieve the mass production of CMS derivatives. Additionally, soybean plants and seedlings were cultivated in the short term in artificial soil (AS) and a Petri dish, respectively, to confirm the existence of the CMS derivatives in soybean.

## Materials and Methods

### Chemicals and Reagents

Daidzein, daidzin, daidzin 6″-*O*-acetate, daidzin 6″-*O*-malonate, genistein, genistin, genistin 6″-*O*-acetate, genistin 6″-*O*-malonate, glycitein, glycitin, glycitin 6″-*O*-acetate, and glycitin 6″-*O*-malonate were purchased from FUJIFILM Wako Pure Chemical Corp (Osaka, Japan). CMS; dimethyl sulfoxide (DMSO); Gelrite™; indole-3-butyric acid (IBA); ascorbic acid; 3-(4,5-dimethylthiazol-2-yl)-2,5-diphenyltetrazolium bromide; methyl jasmonate (MJ); and phosphate-buffered saline were purchased from Sigma-Aldrich Co., LLC (St. Louis, MO, USA). Mass-grade formic acid (FA), acetonitrile, methanol, and water were purchased from Thermo Fisher Scientific (Waltham, MA, USA). The other chemicals employed in this study were of American Chemical Society grade or higher. The Sinhwakong soybean variety (an elite cultivar) was supplied by the National Institute of Crop Science (Cheonju, Republic of Korea).

### Soybean Cultivation Methods

#### AR Culture

**Figure 2:**
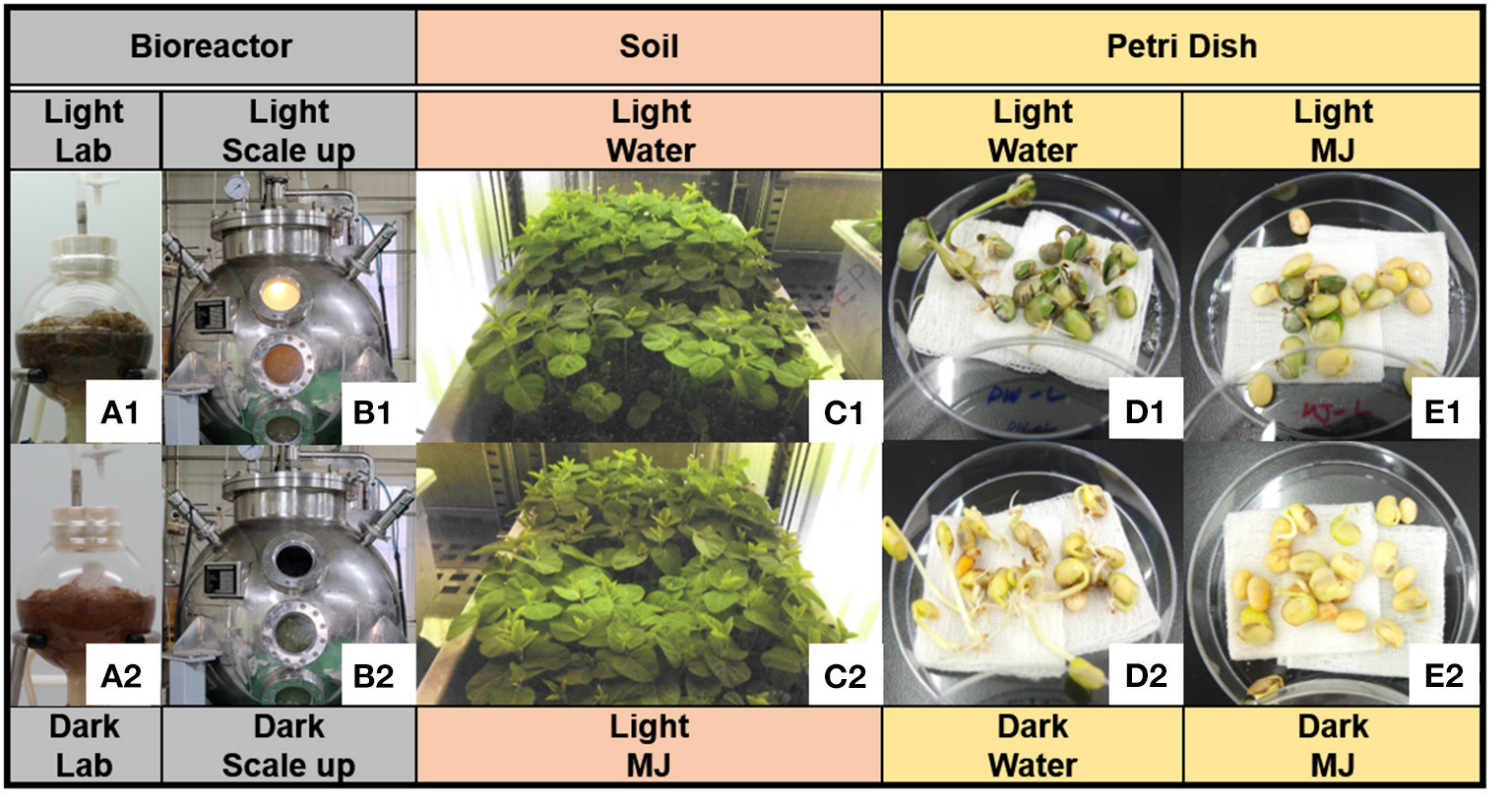
Three cultivation methods of soybean. **(A)** Lab-scale culture of soybean adventitious root (AR) [**(A1)** light condition; **(A2)** dark condition], **(B)** pilot-scale culture of soybean AR, **(C)** soybean plants cultured in artificial soil (AS) with water **(C1)** and methyl jasmonate (MJ) **(C2)** under light–dark cycle conditions, **(D)** seedlings in water under **(D1)** light and **(D2)** dark conditions, and **(E)** seedlings in MJ under **(E1)** light and **(E2)** dark conditions.

The soybean AR culture was performed, following a previous method (Lee et al., 2020). Briefly, the ARs were propagated in a full-strength Murashige and Skoog medium (MSO) (Duchefa, Haarlem, The Netherlands) (Murashige and Skoog, 1962) containing 4 mg/L IBA and 30 g/L sucrose in 3 L bulb-type bubble bioreactors. The bioreactor culture was initiated *via* inoculation with wet ARs at a density of 4.0 g/L and an aeration volume of 0.1 air volume/culture volume per min (vvm) employing an air flow meter (RMA series; Dwyer Instruments, Michigan, USA). The ARs were maintained by subculturing in a fresh liquid medium every 3 weeks under dark conditions at 22 ± 1°C. To increase the phytochemical content, the ARs were cultured under fluorescent light in the bioreactor and with treatment employing a physical elicitor (Yun et al., 2021) for 4 weeks under the aforementioned conditions. Afterward, to increase the phytochemical productivity, the ARs were cultured in a half-strength MSO (2 L) containing 4 mg/L IBA and 30 g/L sucrose under bright light, as well as with treatment employing 50 μM MJ for 5 days before harvesting. The control samples were cultured under the same conditions but in the dark (Figure 2A). AR was mass-produced in a bulb-type bioreactor at an operating capacity of 1,000 L under the same conditions as those in the lab-scale experiment, except for the volume of the half-strength MSO (500 L) and the addition of a fluorescent lamp (Figure 2B). The bright-light conditions were as follows: 60 photosynthetic photon flux density (PPFD) (4,500 lx) and a fluorescent lamp (FHF32W/865; OSRAM GmbH, Munich, Germany). The bright light was supplied for 16 h, followed by 8 h of darkness in a 24 h cycle. After 4 weeks of culturing in the bioreactor, the liquid medium was removed, and the ARs were dried in a convection oven for 24 h at 60°C. The lab-scale and pilot-scale cultures were produced in three batches under the light and dark conditions, respectively.

#### Plant Culture in AS

To compare the chemometric differences between the continuous bioreactor culture and soil cultivation, soybeans were cultured for 5 weeks in AS. The seeds were sterilized with a 2% sodium hypochlorite solution for 20 min. A hundred seeds were sown in a plastic pot (500 × 300 × 200 mm^3^; width × length × height) containing a horticultural substrate (Hanareum; Shinsung Mineral Co., Republic of Korea) and irrigated with tap water (3 L) every 3–4 days, following the overhead irrigation method. The physicochemical characteristics of the horticultural substrate were as follows: pH, 5–7; electrical conductivity, <1.2 dS/m; bulk density, <0.3 g/m^3^; coconut peat, 51.5%; pearlite, 15%; peat moss, 10%; vermiculite, 13%; zeolite, 10%; humic acid, 0.1%; and fertilizer, 0.4%. The light intensity was increased from 70 PPFD (5,200 lx) to a maximum of 200 PPFD (14,800 lx) depending on the growth phase, and the photoperiod was repeated under light (16 h) and dark (8 h) conditions in a 24 h cycle. The plant was cultivated in a growth chamber (GC-1000; JeioTech Co, Daejeon, Republic of Korea) for 5 weeks at a day/night temperature and humidity of 23 ± 2°C and 50 ± 5%, respectively (Figure 2C). To increase the phytochemical content of the roots, 50 μM MJ (2 L) in water was applied *via* the bottom irrigation method 5 days before harvesting. The plants, which were utilized as the controls, were supplied by regular tap water rather than MJ. After 5 weeks of cultivation, the grown plants were uprooted whole, and the accompanying soil particles were removed using running water. Thereafter, the roots were dried for 24 h at 60°C.

#### Germination of Seedlings in a Petri Dish

The soybeans were washed two times and soaked in pure water for 15 h. Thereafter, the soaked soybeans were sterilized with 70% (v/v) aqueous ethyl alcohol for 10 s and washed with pure water. Twenty prepared seeds were loaded onto a Petri dish (in triplicates) and covered with a four-layered gauze sheet. Pure water (10 mL) or 10 μM MJ was added to each Petri dish as a growth medium. A set of growth media was cultured without light, while the other set was exposed to light (10,000 lx). The Petri dishes were placed in a plant growth chamber (GC-1000; JeioTech Co) at a temperature of 20°C and relative humidity of 40%. Next, pure water (5 mL) was added to the Petri dish after three culture days (Figures 2D,E). The seedlings were collected after three, six, and nine culture days and freeze-dried *via* immersion in liquid nitrogen.

#### Purification and Identification of the CMS Derivatives

Powdery cultured soybean AR was dissolved in 60% (v/v) aqueous methanol (50 mg/mL) and sonicated for 20 min. The methanolic extracts were centrifuged for 10 min at 4,000 × g and 25°C, after which the supernatants were filtered through a 0.45 μm polyvinylidene fluoride (PVDF) syringe filter (Pall Corp., Port Washington, NY, USA). The filtered solutions were injected (200–1,000 μL per injection) into a preparative high-performance liquid chromatography (HPLC) column that was equipped with a 172-diode array detector, 321 binary solvent pump, and GX-271 liquid handler (Gilson Inc., Middleton, WI, USA). Two fractions (F1 and F2) were collected for a single peak under programmed elution conditions employing a preparative separation column (ZORBAX Eclipse XDB C_18_, 80 Å, 5 μm, 21.2 × 150 mm; Agilent Technologies, Santa Clara, CA, USA) at 30°C with a column heater (CO-2060; JASCO Corp., Tokyo, Japan). Each fraction in the repeated injections was combined, evaporated, and freeze-dried. The details of the preparative elution program and peak collection are presented in Supplementary Figure S1.

The compounds in the purified fractions were identified *via* quadrupole time-of-flight high-resolution electrospray ionization mass spectrometry (Q-TOF-HR ESI/MS). An UltiMate 3000 (Thermo Fisher Scientific), which was equipped with a CORTECS C_18_ column (90 Å, 1.6 μm, 2.1 mm × 100 mm; Waters Corp., Milford, MA, USA), was employed for the separation. The column temperature and flow rates were 40°C and 0.5 mL/min, respectively. The mobile phase comprised 0.1% (v/v) FA in water (Solvent A) and 0.1% (v/v) FA in acetonitrile (Solvent B). Further, the following linear gradient was applied: 86% A/14% B at 0 min, 86% A/14% B at 1.5 min, 74% A/26% B at 3 min, 74% A/26% B at 5 min, 20% A/80% B at 5.5 min, 86% A/14% B at 6 min, and 86% A/14% B at 7 min. The precursors and fragments were mass-detected using a Triple TOF 5600+ System (AB Sciex LLC, Framingham, MA, USA) under the following conditions: ionization mode, positive and negative; MS scan type, full scanning and information-dependent acquisition scanning; ionization source, ESI; MS scan range, 200–2,000 *m/z*; MS/MS scan range, 30–2,000 *m/z*; nebulizing gas pressure (ion source 1), 50 psi; heating gas pressure (ion source 2), 50 psi; curtain gas pressure, 25 psi; desolvation temperature, 500°C; ion spray voltage floating, 5.5 kV (positive) and 4.5 kV (negative); declustering potential (DP), 60 (positive) and −60 (negative); collision energy (CE), 10 (positive) and −10 (negative); CE, 35 ± 15 (positive) and −35 ± 15 (negative); and collision gas, He. The purity of the compounds was tentatively calculated *via* a spectral purity check employing an ACQUITY photodiode array detector (PDA) and the Empower 3 software (Waters Corp.) (Fabre et al., 1995; Singh et al., 2012). Each of the prepared compounds was dissolved in 500 μL of DMSO-*d*_6_, after which one-dimensional (1D) (^1^H and ^13^C) and two-dimensional (2D) [heteronuclear single-quantum coherence (HSQC) and heteronuclear multiple bond correlation (HMBC)] analyses were performed using a high-resolution nuclear magnetic resonance (NMR) spectrometer (600 MHz) (AVANCE 600; Bruker, Billerica, MA, USA). The structures of the compounds were determined using the Mnova 11 software (Mestrelab Research, Santiago de Compostela, A Coruña, Spain).

### Determination of the Chemical Composition

The freeze-dried ARs, the plants from AS, and the soybean seedlings were pulverized using a Tubemill™ (IKA, Staufen, Germany) for 1 min at 25,000 rpm. The powdered samples (50 mg each) were mixed in 1.0 mL of 10% (v/v) DMSO in methanol, followed by 10 min of sonication. Thereafter, they were properly diluted using 10% (v/v) DMSO in methanol. The diluted samples were filtered through a 0.45 μm PVDF syringe filter (Pall Corp.).

The contents of the 12 isoflavones, CMS, and purified flavonol glycosides in the samples were analyzed using an ACQUITY UPLC system that was equipped with a binary pump, PDA detector, and mass detector (QDa™; Waters Corp.), as well as a CORTECS C_18_ column (90 Å, 1.6 μm, 2.1 × 100 mm; Waters Corp.). The flow rate, column temperature, and injection volume were 0.5 mL/min, 30°C, and 1 μL, respectively. Gradient elution was performed employing solvents A and B (the aforementioned linear gradient was applied).

The optimal conditions for mass detection, as well as the validated quantification method, have been described in the literature (Rha et al., 2021). The CMS monoglucoside (coumestrin; CMSN) and malonyl CMSN (M-CMSN) contents of the samples were analyzed employing the aforementioned instrument in different injection sets at a cone voltage of 5 V in the positive acquisition mode. The data were collected and processed using Empower 3 (Waters Corp.).

### Statistical Analysis

Data are expressed as the mean ± standard error of the mean for a set of five plants and a set of three extracts. One-way analysis of variance was performed using comparisons of each pair by Student's *t*-test or comparisons for all pair by Tukey-Kramer's honestly significant difference test at an α = 0.05 level using JMP 16 (SAS Institute Inc., Cary, NC, USA).

## Results

### Biomass Obtained *via* the Three Cultivation Methods

Employing the 3 L and 1,000 L bioreactors, the yields of the dried ARs were ~4.0 and 3.0 g/L, respectively. The lengths of the shoots and roots of the AS-grown control and test (elicitor) plants were similar, in the ranges of 250–300 and 200–300 mm, respectively. The dry weights of the shoots and roots of the control culture were 5.21 ± 0.77 and 0.59 ± 0.11 g/10 plants, respectively, and those of the test culture were 4.97 ± 0.38 and 0.51 ± 0.04 g/10 plants, respectively. The dry weights of the shoots and roots of the control and elicitor cultures were not significantly different (*p* < 0.05). The initial dry weight of a soybean seedling was 2.75 ± 0.04 g/20 beans, while that of the 3-day seedling decreased to 2.06 ± 0.03 g/20 beans in the four germination groups. The dry weights reduced after germination for all the treatments and stagnated during the water and light treatments, while those for the other treatments decreased during the 9-day culture period (Supplementary Figure S2).

### Newly Identified CMS Derivatives

AR cultivation availed substantial CMSN derivatives along with considerable root masses. Moreover, different chromatograms, which indicated the presence of the different molecules, were observed. Further, we observed two major CMSN derivatives, including their isomers. Here, we elucidated their structures.

#### Confirmation of the Structure of F1

**Figure 3:**
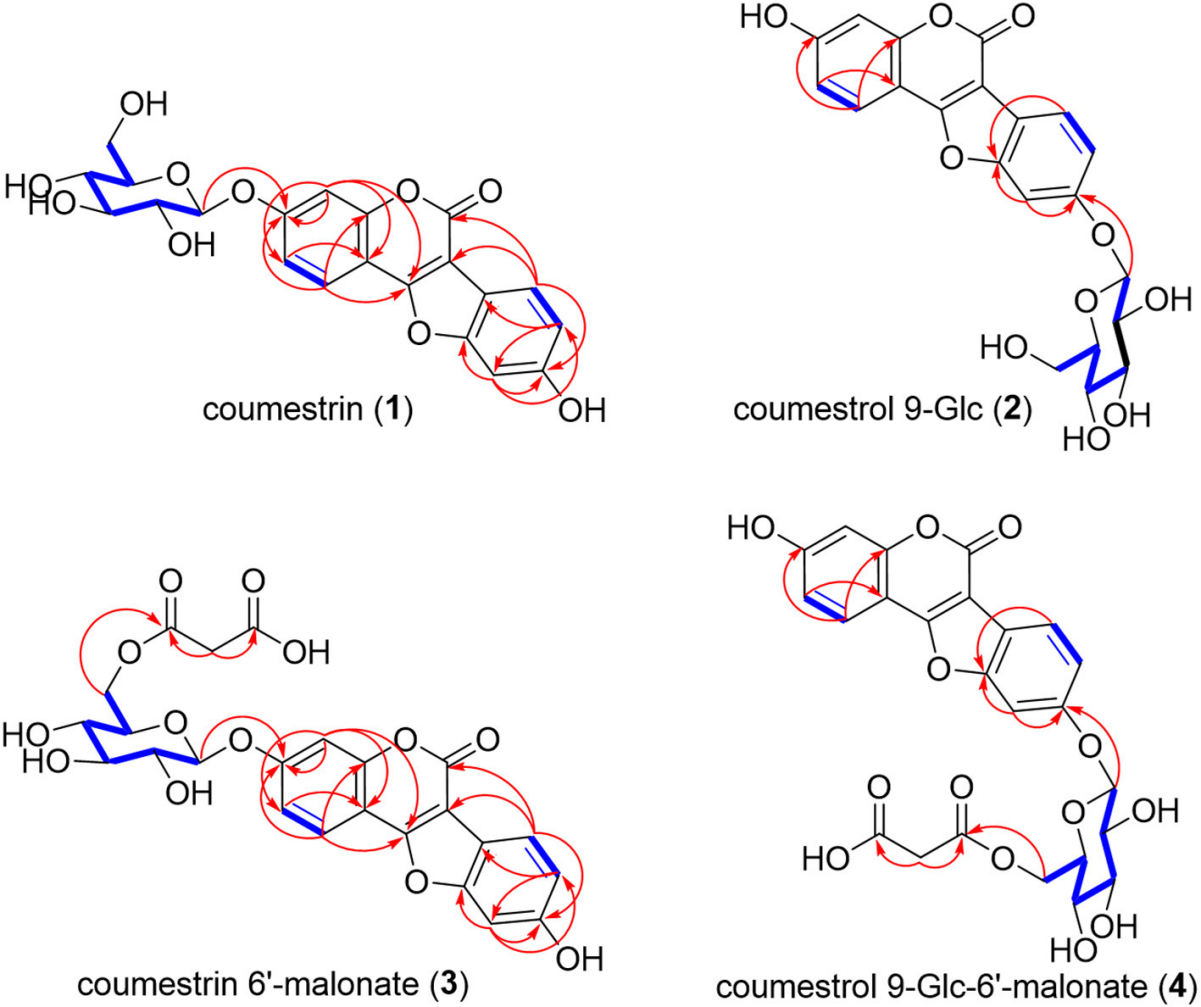
COSY and HMBC for the structural interpretations of coumestrin (CMSN), malonyl CMSN (M-CMSN), and the isomers. ^1^H–^1^H coupling and ^1^H–^13^C long-range correlations observed in the COSY and HMBC spectra of CMSN **(1)**, CMS 9-Glc **(2)**, CMSN 6′-malonate **(3)**, and CMS 9-Glc-6′-malonate **(4)**, respectively. The bold blue lines indicate the coupling correlations between the proton and proton signals in the COSY spectrum, and the red arrows show the *J*_2_ and *J*_3_ correlations between the proton and carbon signals in the HMBC spectrum.

The molecular formula of F1, as determined by Q-TOF-HR ESI/MS [positive [M + H]^+^, *m/z* 431.0969 (calculated (calc.) for C_21_H_19_${\text{O}}_{10}^{+}$, 431.0973) and negative [M – H]^−^, *m/z* 429.0829 (calc. for C_21_H_17_${\text{O}}_{10}^{-}$, 429.0827)], was C_21_H_18_O_10_ (Table 1, Supplementary Figure S3). The ^1^H-NMR spectrum (600 MHz, DMSO-*d*_6_) revealed the presence of four 1,2,4-trisubstituted benzene-ring moieties and two hemiacetal signals; we confirmed *via* the integral of ^1^H-NMR that the ratio of major to minor components was 8:1 (Supplementary Figure S4). The major-component signals were detected at [δ_H_ 7.98 (1H, d, *J* = 9.0 Hz, H-1), δ_H_ 7.27 (1H, d, *J* = 2.4 Hz, H-4), and δ_H_ 7.17 (1H, dd, *J* = 9.0, 2.4 Hz, H-2)] and [δ_H_ 7.75 (1H, d, *J* = 8.4 Hz, H-7), δ_H_ 7.21 (1H, d, *J* = 1.8 Hz, H-10), and δ_H_ 6.99 (1H, dd, *J* = 8.4, 1.8 Hz, H-8)] corresponding to the two 1,2,4-trisubstituted benzene ring moieties and at δ_H_ 5.09 (1H, d, *J* = 6.6 Hz, H-1′) as a hemiacetal signal, as well as at δ_H_ 3.73 (1H, br d, *J* = 11.4 Hz, H-6′b) and δ_H_ 3.48 (1H, dd, *J* = 11.4, 4.2 Hz, H-6′a) corresponding to the oxymethylene signal and δ_H_ 3.46 (1H, br dd, *J* = 8.4, 4.2 Hz, H-5′), δ_H_ 3.31 (1H, overlapped, H-3′), δ_H_ 3.30 (1H, overlapped, H-2′), and δ_H_ 3.31 (1H, overlapped, H-3′) corresponding to four oxygenated methine signals, respectively (Table 2). The ^1^H-NMR spectrum revealed two 1,2,4-trisubstituted benzene ring moieties of minor components at [δ_H_ 7.90 (1H, d, *J* = 8.4 Hz, H-1), δ_H_ 7.21 (1H, d, *J* = 1.8 Hz, H-4), and δ_H_ 6.99 (1H, dd, *J* = 8.4, 1.8 Hz, H-2)] and [δ_H_ 7.75 (1H, d, *J* = 8.4 Hz, H-7), δ_H_ 7.21 (1H, d, *J* = 1.8 Hz, H-10), and δ_H_ 6.99 (1H, dd, *J* = 8.4, 1.8 Hz, H-8)], as well as a minor anomeric proton at δ_H_ 5.09 (1H, d, *J* = 6.6 Hz, H-1′). The ^13^C-NMR spectrum (150 MHz, DMSO-*d*_6_, δ_C_) mainly exhibited 21 carbon signal components along with weak minor peaks, which confirmed that F1 comprised a phenolic compound and sugar (Supplementary Figure S5). Regarding the major component, a carbonyl carbon signal (δ_C_ 158.0), five oxygenated quaternary carbon signals (δ_C_ 160.5, 159.4, 157.8, 156.7, 154.6), three olefinic quaternary carbon signals (δ_C_ 114.7, 107.0, 103.8), and six olefinic methine carbon signals (δ_C_ 122.9, 121.3, 114.9, 114.8, 104.6, 99.2) were detected as the signals of an aglycone, which was identified as a CMS. The carbon signals of the minor component were detected as very weak signals at δ_C_ 162.4 (C-3), 160.0 (C-11a), 157.6 (C-5), 157.5 (C-10a), 155.1 (C-4a), 123.8 (C-1), 121.3 (C-7), 117.6 (C-2), 117.5 (C-8), 115.0 (C-6b), 104.6 (C-11b), 103.8 (C-4), 100.6 (C-10), 102.0 (C-6a), and 100.6 (C-10). The chemical shifts of two anomeric carbons (δ_C_ 101.6 and 101.7 corresponding to the major and minor components, respectively) overlapped with eight oxymethine (δ_C_ 77.6, 76.9, 73.6, 70.1) and two oxymethylene (δ_C_ 61.1) carbon signals (Table 2, Supplementary Figure S5). The HMQC spectrum enabled the identification of the proton and carbon signals of the corresponding major and minor protons, respectively (Supplementary Figure S6). The aforementioned ^1^H- and ^13^C-NMR, as well as HMQC data, indicated that F1 was a mixture (8:1) of a glycosylated CMS and its analog. CMS comprising two 1,2,4-trisubstituted benzene ring moieties and sugar was constructed by its correlation spectroscopy (COSY) and HMBC (Figure 3, Supplementary Figures S7, S8). The COSY spectrum revealed the presence of glucopyranose [δ_H_ 5.09 (H-1′) or δ_H_ 5.00 (H-1′), δ_H_ 3.30 (H-2′), δ_H_ 3.31 (H-3′), δ_H_ 3.19 (H-4′), δ_H_ 3.46 (H-5′), δ_H_ 3.48 (H-6′a), and δ_H_ 3.73 (H-6′b)]; the coupling constants (*J* = 6.6 Hz) of δ_H_ 5.09 (H-1′) and (*J* = 7.8 Hz) of δ_H_ 5.00 (H-1′) confirmed the presence of an anomeric hydroxy group exhibiting a β-configuration. Regarding the major component, the COSY of H-1 (δ_H_ 7.98) with H-2 (δ_H_ 7.17)/H-7 (δ_H_ 7.75) with H-8 (δ_H_ 6.99), as well as HMBC from H-1 to C-11b (δ_C_ 107.0); H-2 to C-3 (δ_C_ 160.5), C-4a (δ_C_ 159.4), and C-11a (δ_C_ 154.6); H-4 (δ_H_ 7.27) to C-2 (δ_C_ 114.9), C-11a, and C-11b; H-7 to C-6 (δ_C_ 158.0), C-6a (δ_C_ 103.8), and C-9 (δ_C_ 156.7); H-8 to C-6b (δ_C_ 114.7) and C-10 (δ_C_ 99.2); and H-10 (δ_H_ 7.21) to C-8 (δ_C_ 114.8), C-9 (δ_C_ 156.7), and C-10a (δ_C_ 157.8) confirmed that the CMS skeleton was a major component. HMBC of a methylene H-1′ (δ_H_ 5.09) with an oxygenated quaternary carbon C-3 (δ_C_ 160.5) corresponding to CMS 3-*O*-β-d-glucopyranose (CMSN, **1**) indicated the major component (Figure 3, Supplementary Figure S8). In the gHMBC spectrum of the minor component (Supplementary Figure S9), the signal of the minor anomeric proton (δ_H_ 5.00, H-1′) exhibited the cross-peak with the signal of the oxygenated olefinic quaternary carbon (δ_C_ 156.5, C-9); the long-range correlations of H-10 (δ_H_ 7.56) with C-9 and C10a (δ_C_ 157.5) and of H-7 (δ_H_ 7.80) with C-10a confirmed that the *O*-β-d-glucopyranose was positioned at C-9 as a novel compound. A previous ^1^H-NMR report of CMS confirmed that H-1, H-2, H-4, H-7, H-8, and H-10 were detected at δ_H_ 7.85, 6.93, 6.91, 7.69, 6.95, and 7.17 in a DMSO-*d*_6_ solvent, respectively (Sheng et al., 2016). Three H-1, H-2, and H-4 CMCN (CMS 3-Glc, **1**) signals were observed as major components at δ_H_ 7.98, 7.17, and 7.27, respectively, with down-shifted values corresponding to the glycosylation effect. Similarly, H-7, H-8, and H-10 signals were observed at δ_H_ 7.80, 7.19, and 7.56, respectively, and were downshifted because of the position of *O*-β-d-glucopyranose to C-9 of CMS 9-*O*-β-d-glucopyranose (**2**), another novel compound. Overall, F1 was identified as CMS 3-*O*-β-d-glucopyranose (CMSN, **1**), a major component, and CMS 9-*O*-β-d-glucopyranose (CMS 9-Glc, **2**).

**Table 1:** Mass identification of the purified coumestrin (CMSN) derivatives.

**Comp.^†^**	**Positive**			**Negative**			**Formula (M)**	**Identification**
	**MS1^**a**^**	**Error ppm**	**MS2^**b**^**	**MS1^**a**^**	**Error ppm**	**MS2^**b**^**		
F1	431.0969	−0.9	269.0447	429.0829	0.5	267.0298	C_21_H_18_O_10_	CMSN analog^c^
						239.0349		
F2	517.0972	−0.9	453.3214	515.0833	0.4	429.0827	C_24_H_20_O_13_	CMSN 6′*-*malonate analog^d^
			377.0616			375.0915		
			304.0946			267.0304		
			269.0447			239.0351		

† *Purified compounds, F1 and F2, indicate the fractions from preparative HPLC. Refer to Supplementary Figure S1*.

a *Molecular mass of the precursors*.

b *Molecular mass of the molecular fragments*.

c *CMSN (coumestrol (CMS) 3-O-β-d-glucopyranoside) and CMS 9-Glc (CMS 9-O-β-d-glucopyranoside)*.

d *CMSN 6′-malonate (CMS 3-O-β-d-(6′-O-malonyl)-glucopyranoside) and CMS 9-Glc-6′-malonate (CMS 9-O-β-d-(6′-O-malonyl)-glucopyranoside)*.

**Table 2:** NMR analyses of the newly identified CMSN derivatives.

**No**.	**CMSN (1)**		**CMS 9-Glc (2)**		**CMSN 6** ^**′**^ **-malonate (3)**		**CMS 9-Glc-6** ^**′**^ **-malonate (4)**	
	**δ_H_ (*J* in Hz)**	**δ_C_**	**δ_H_ (*J* in Hz)**	**δ_C_**	**δ_H_ (*J* in Hz)**	**δ_C_**	**δ_H_ (*J* in Hz)**	**δ_C_**
1	7.98, d, 9.0	122.9	7.90, d, 9.0	123.8	7.98, d, 9.0	122.9	7.92, d, 9.0	123.2
2	7.17, dd, 9.0, 2.4	114.9	6.96, dd, 9.0, 2.4	117.6	7.17, dd, 9.0, 2.4	114.8	6.95, dd, 9.0, 2.4	114.5
3	–	160.5	–	162.4	–	160.3	–	162.6
4	7.27, d, 2.4	104.6	6.93, d, 2.4	103.8	7.23, d, 2.4	104.6	6.92, d, 2.4	103.8
4a	–	159.4	–	155.1	–	159.3	–	155.4
6	–	158.0	–	157.6	–	158.0	–	157.6
6a	–	103.8	–	102.0	–	103.8	–	102.0
6b	–	114.7	–	115.0	–	114.7	–	114.7
7	7.75, d, 8.4	121.3	7.80, d, 8.4	121.3	7.75, d, 8.4	121.3	7.81, d, 8.4	121.3
8	6.99, dd, 8.4, 1.8	114.8	7.19, dd, 8.4, 1.8	117.5	6.99, dd, 8.4, 1.8	114.7	7.15, dd, 8.4, 1.8	114.9
9	–	156.7	–	156.5	–	156.7	–	156.5
10	7.21, d, 1.8	99.2	7.56, d, 1.8	100.6	7.21, d, 1.8	99.2	7.55, d, 1.8	100.4
10a	–	157.8	–	157.5	–	157.8	–	158.0
11a	–	154.6	–	160.0	–	154.6	–	162.0
11b	–	107.0	–	104.6	–	107.1	–	104.8
1′	5.09, d, 6.6	100.6	5.00, d, 7.8	101.7	5.11, d, 7.2	100.2	5.21, d, 7.8	100.2
2′	3.30, overlapped	73.6	3.30, overlapped	73.6	3.33, overlapped	73.6	3.33, overlapped	73.5
3′	3.31, overlapped	76.9	3.31, overlapped	76.9	3.37, overlapped	76.7	3.37, overlapped	76.6
4′	3.19, dd, 8.4, 8.4	70.1	3.19, dd, 8.4, 8.4	70.1	3.23, dd, 8.4, 8.4	70.3	3.23, dd, 8.4, 8.4	70.1
5′	3.46, br dd, 8.4, 4.2	77.6	3.46, br dd, 8.4, 4.2	77.6	3.74, br dd, 8.4, 6.6	74.4	3.74, br dd, 8.4, 6.6	74.3
6′	3.48, dd, 11.4, 4.2	61.1	3.48, dd, 11.4, 4.2	60.9	4.12, dd, 12.0, 6.6	63.9	4.12, dd, 12.0, 4.2	64.0
	3.73, br. D, 11.4		3.73, br. D, 11.4		4.35, br. D, 12.0		4.35, br. D, 12.0	
1″					–	170.7	–	170.7
2″					2.07, s	21.1	2.07, s	21.1
3″					–	170.7	–	170.7

^1^*H- and ^13^C-NMR data of the compounds [δ_H_ (600 MHz), δc (150 MHz) in ppm, coupling pattern, J in Hz]*.

*Data were measured in DMSO-d_6_*.

#### Confirmation of the Structure of F2

The Q-TOF-HR ESI/MS data of the positive molecule [M + H]^+^ at *m/z* 517.0972 (calc. for C_24_H_21_${\text{O}}_{13}^{+}$, 517.0977), as well as the negative molecule [M – H]^−^ at *m/z* 515.0833 (calc. for C_24_H_19_${\text{O}}_{13}^{-}$, 515.0831) confirmed that the molecular formula of F2 was C_24_H_20_O_13_ (Table 1, Supplementary Figure S3). The ^1^H-NMR (600 MHz, DMSO-*d*_6_) and ^13^C-NMR (150 MHz) spectra of F2 were almost similar to those of F1, except for the presence of one additional malonate and two extra carbonyl carbon (δ_C_ 170.7, δ_C_ 170.7) and extra methylene (δ_H_ 2.07; δ_C_ 21.1) signals (Table 2, Figure 2, and Supplementary Figures S9, S10). The mass spectrometry and 1D NMR analyses revealed that the structure of F2 corresponded to CMSN containing malonate. Moreover, we observed that the ratio of the major component to the minor one of each peak integral value of ^1^H-NMR was 8:1, corresponding to the ratio of CMSN (**1**) to coumestrol 9-Glc (**2**) in F1 (Supplementary Figure S9). The HMQC spectrum revealed the identities of the proton and carbon signals, as well as their corresponding protons (Table 2, Supplementary Figure S11). In the gHMBC spectrum, H-6′a (δ_H_ 4.12) and H-6′b (δ_H_ 4.35), which were downshifted *via* carbonylation, exhibited correlations with C-1″ (δ_C_ 170.7), and H-2″ (δ_H_ 2.07) exhibited correlations with C-1″ and C-3″ (δ_C_ 170.7) (Figure 3, Supplementary Figure S12), indicating that a malonate group was positioned at C-6′ of glucopyranose. HMBC and COSY indicated that the major and minor components of F2 were CMSN 6′-malonate (**3**) and CMS 9-*O*-β-d-(6′-*O*-malonyl)-glucopyranose [CMS 9-Glc-6′-malonate, (**4**)], respectively (Figure 3, Supplementary Figures S12, S13). Thus, **4** of F2 was identified as a novel compound.

### Chemical Composition of the Derivatives Obtained *via* Cultivation

Based on the area under the curve of the maximum ultraviolet (UV) absorbance (220–460 nm) (Supplementary Figures S14, S15), the purities of F1 and F2, as obtained *via* peak collection employing preparative HPLC, were 70% and 64%, respectively. The purities of F1 and F2 were exploited to quantify the CMSN and M-CMSN contents of all the samples, respectively.

**Figure 4:**
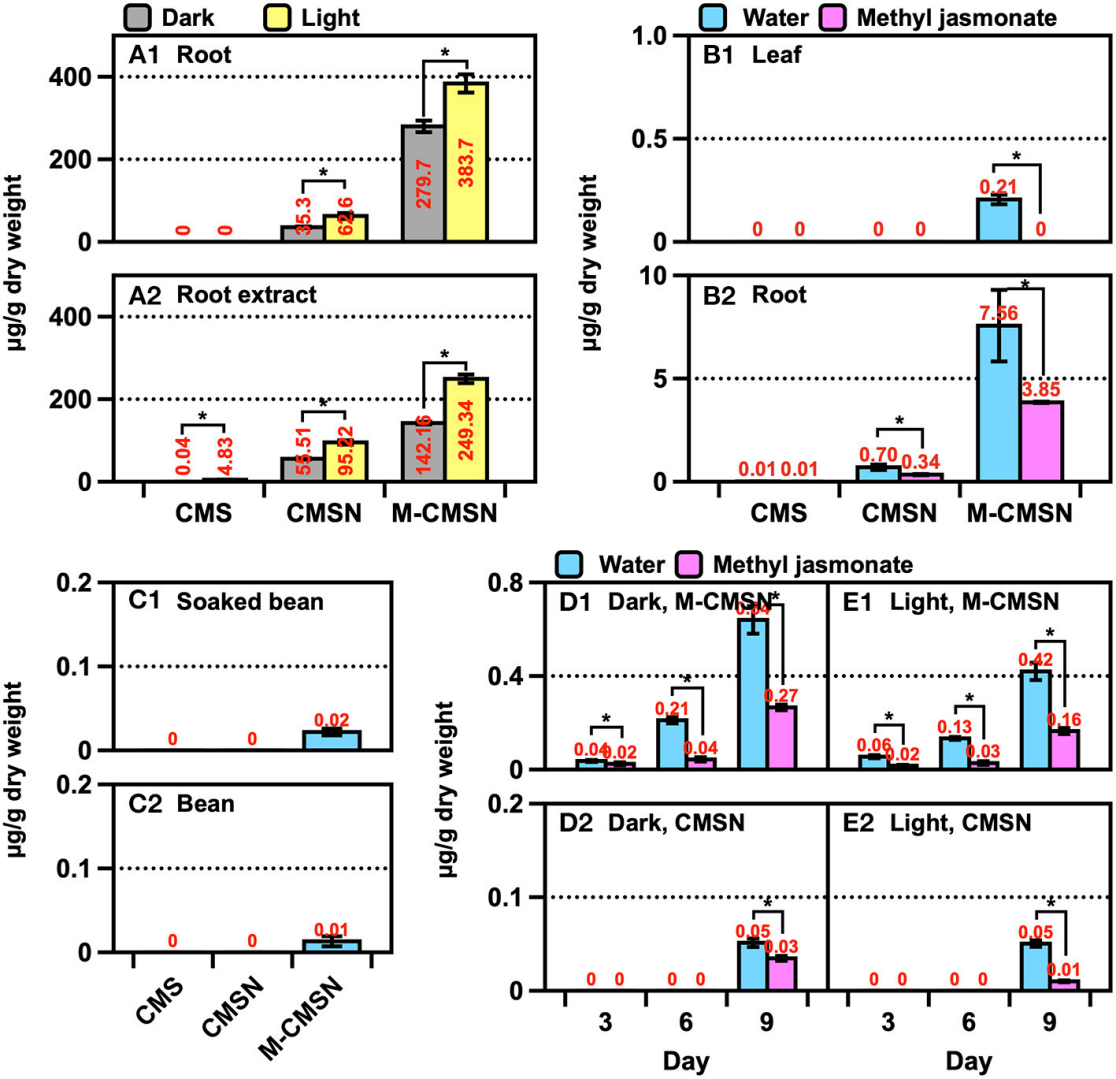
Changes in the CMSN and M-CMSN contents of **(A)** soybean AR, **(B)** soybean plants cultivated in artificial soil (AS), **(C)** untreated and soaked soybean, and **(D,E)** soybean seedlings. The data are expressed as the mean ± standard error of the mean. Asterisk “*” on the bar indicates significant differences of the comparisons for each pair by Student's *t*-test at an α = 0.05 level.

The CMS derivatives were present in the ARs, beans, leaves, roots, and seedlings of the soybean (Figure 4). A significant amount of M-CMSN (~400 μg/g) was produced in the ARs, while a four-fold-lower quantity of CMSN was produced under light (Figure 4A1). Under dark conditions for the AR culture, 0.75-fold-lower amounts of the CMS derivatives were obtained than the amounts under the light conditions. Notably, CMS was not detected in the ARs, although its traces were detected in the AR extract owing to the degradation of the CMS derivatives at the extraction stage (Figure 4A2). Similarly, M-CMSN was obtained in a 10-fold-higher amount (7.6 μg/g) in the roots than in the leaves (Figure 4B). Compared with the utilization of the control (water) in AS, the MJ treatment could not effectively increase the production of the CMS derivatives (Figure 4B2). Lower amounts of M-CMSN were detected in the untreated and soaked soybeans (Figure 4C). The contents of the CMS derivatives in the soybean seedlings increased gradually with the cultivation period (Figures 4D,E). The M-CMSN content of the soybean seedling was 8–14-fold higher than the CMSN content. Thus, darkness and water contributed majorly to the production of M-CMSN (Figures 4D,E). Compared with the results for AR, the CMS derivative contents of the plants and seedlings were substantially lower.

**Figure 5:**
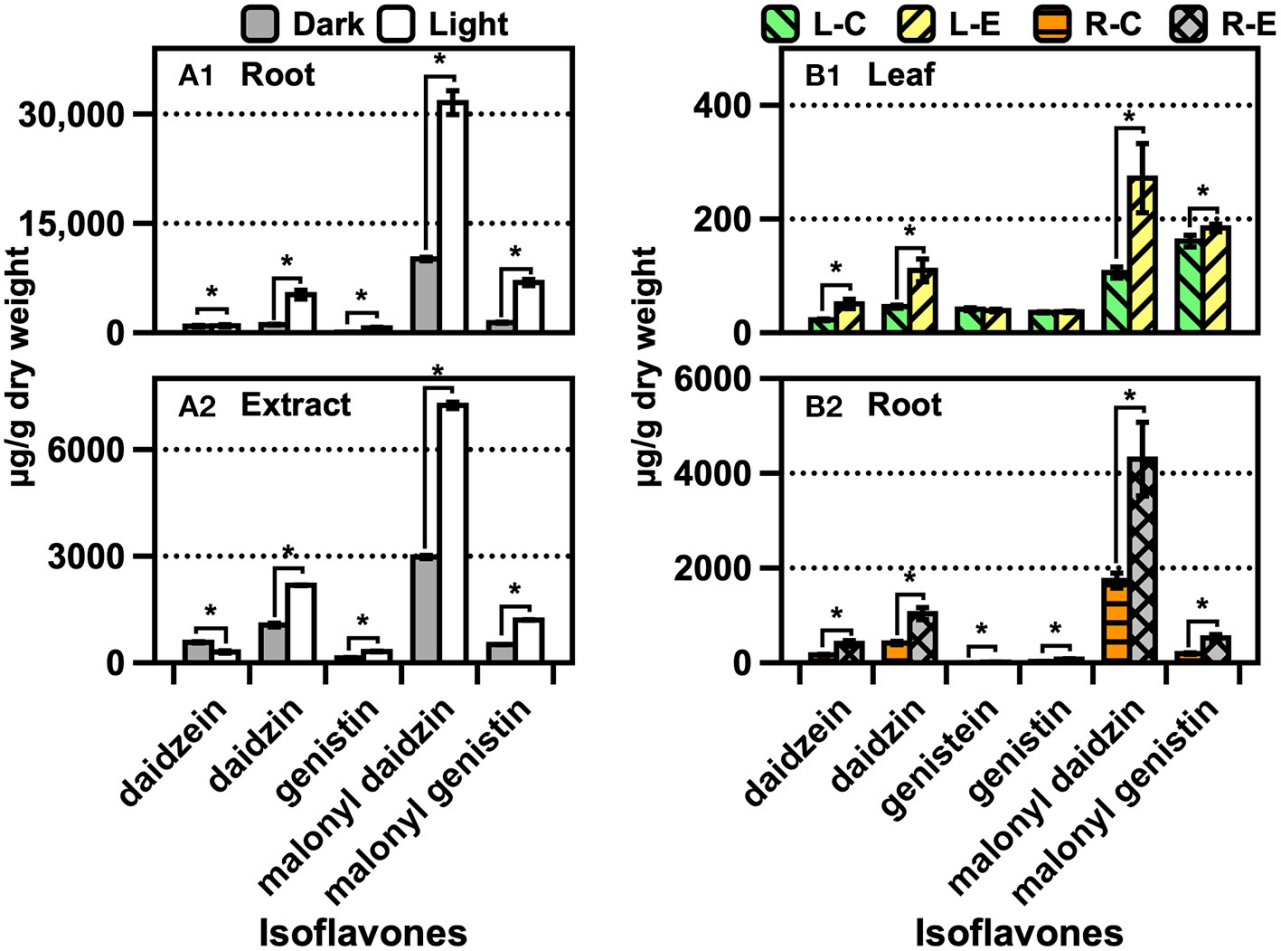
Contents of isoflavones and kaempferol glycosides of **(A)** soybean AR and **(B)** soybean plants cultivated in AS. The data are expressed as the mean ± standard error of the mean. Asterisk “*” on the bar indicates the significant differences of the comparisons for each pair by Student's *t*-test at an α = 0.05 level.

**Figure 6:**
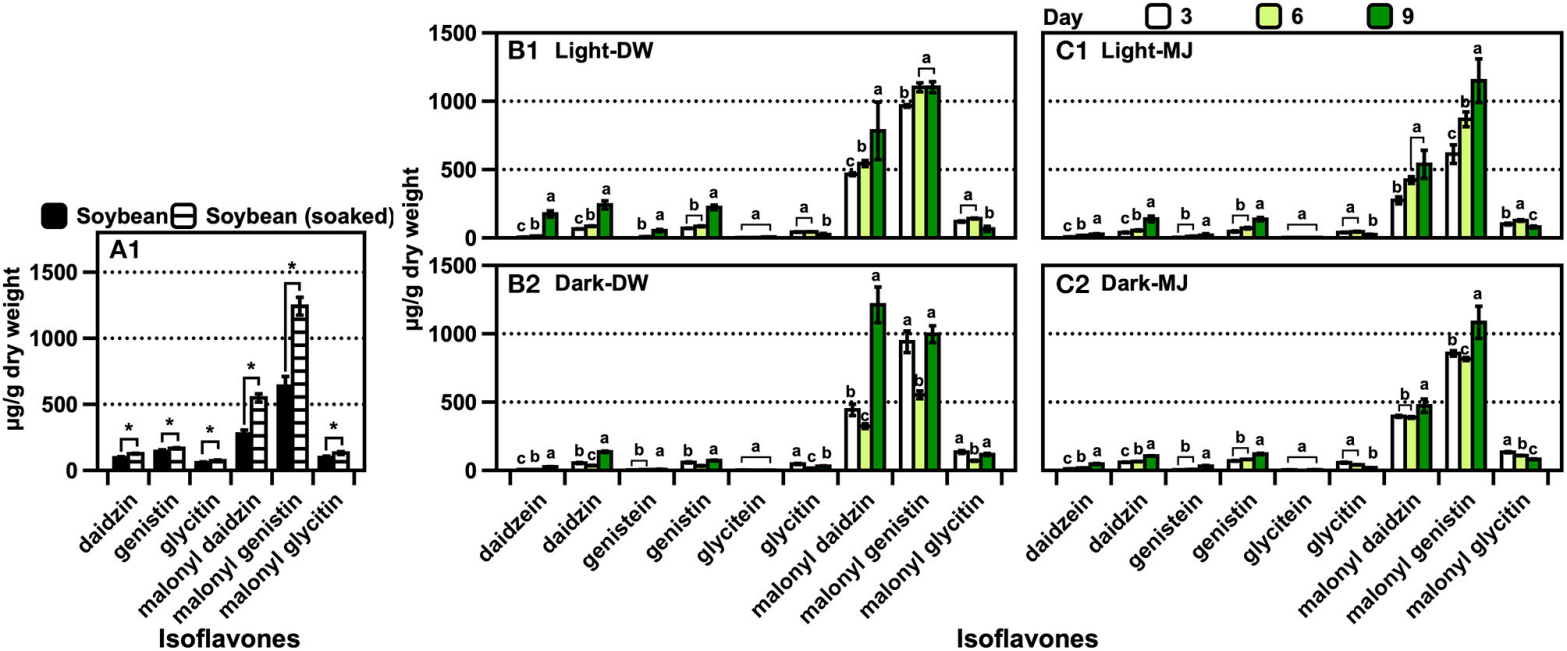
Isoflavone contents of **(A)** soybean before germination, **(B)** soybean seedlings in water, and **(C)** soybean seedlings in MJ solution. The data are expressed as the mean ± standard error of the mean. Asterisk “*” on the bar indicates the significant differences of the comparisons for each pair by Student's *t*-test at an α = 0.05 level. The lowercase letters on the bar in the same compounds indicate the significant differences of the comparisons for all the pairs by the Tukey–Kramer honestly significant difference test at an α = 0.05 level.

The trend of the isoflavone content was similar to that of the CMS derivatives across the samples (Figures 5A,B). A significant amount of malonyl daidzin was produced from different isoflavones in AR, and over a two-fold higher amount was obtained under light (Figure 5A). Although the amounts of the daidzin and malonyl genistin accounted for the second-largest, acetylated isoflavones were undetected. The isoflavone composition of the plants was similar to that of ARs, while that of the leaves was 10-fold lower than that of the roots (Figure 5B). The chemical elicitor treatment afforded an approximately two-fold increase in the isoflavone content compared with that in the control (water). The kaempferol glycoside contents of the leaves obtained *via* the control or MJ treatments did not differ (Supplementary Figure S15). The contents of isoflavones (of which malonyl genistin accounted for the major component) of the soybean increased significantly (*p* < 0.05) by approximately 1.9-fold after soaking in water (Figure 6A1). The isoflavone content of the seedlings increased greatly across the growth days [the only difference in the malonyl isoflavone content was observed in the 9 days of darkness–water treatment (Figure 6B2)], and the isoflavone contents were not significantly different among the other treatments (Figures 6B,C). The kaempferol glycoside content of the seedlings increased significantly, following the illumination treatments (Supplementary Figure S15); moreover, the MJ treatment could hinder the production of kaempferol glycosides because it inhibited the growth of the seedlings.

## Discussion

### Culture Methods

The difference between the yields obtained employing the lab-scale (4 g/L) and pilot-scale (3 g/L) bioreactors was mainly because of interferences, such as the low mass-transfer rate, hydraulic pressure, and shear stress of the large-scale reactor (Murthy et al., 2014), which reduced the yield. Thus far, no significant correlation has been calculated, thereby necessitating further optimization needs to scale up the productivity. The reproducible AR cultivation approach reported herein could represent a milestone in this research area and is more effective than the large-scale cultivation of plant cells, which is rarely adopted because of its many associating limitations (Lee et al., 2014). The dry weight yield of the root in the AS culture was ~0.2 g/L soil, which was lower than that of the root cultivated in the lab-scale bioreactor (4 g/L).

### Newly Identified M-CMSN

The difference between the yields of the two bioreactors was mainly because of their light transparency. AR in the 3 L glass bioreactor was evenly exposed to the light source, and aeration facilitated the sufficient movement of the AR during cultivation (3 L bioreactor for 4 weeks; Supplementary Video S1). Conversely, AR in the 1,000 L stainless bioreactor was not fully exposed to the light source owing to the limitation of the light reflex and penetration (the light source was anchored on a fixed spot). Nevertheless, the periodic movement of AR in the bioreactor *via* aeration might have triggered the synthesis of phytoalexins, following light stress. It is well-known that the synthesis of CMSN can be facilitated by environmental factors and periodic changes in the plant growth cycle (Song et al., 2014; Mun et al., 2021). To date, only Romani et al. (2003) have detected M-CMSN in soybean roots *via* a tentative assignment by the UV spectra and mass fragmentation only. Additionally, Oshima et al. (2016) performed the structural analysis of M-CMSN in a soybean seed coat *via* NMR. Therefore, there are no definite evidence regarding the presence of M-CMSN, which contains a relatively unstable malonyl moiety, in soybean root. M-CMSN is produced from malonyl daidzin, which is abundant in soybean roots, *via* an intrinsic biological transformation against biotic and abiotic stresses. Employing the cultivation method, M-CMSN was detected in the roots and leaves of soybean; a small fraction of CMSN was also detected. However, CMS, an aglycone form of CMSN, was only detected in the root extract owing to its degradation during heating. Therefore, unlike the presence of CMS in a late-harvested soybean leaf, CMS might not be produced *via* the intrinsic metabolism of the root. To effectively produce M-CMSN, AR cultivation is preferred to the AS planting and Petri dish seedling methods. This indicates that field-grown roots might not be an alternative material for synthesizing these novel compounds. Furthermore, our results demonstrated the presence of CMS 9-glucoside and CMS 9-glucoside-6′-malonate, which could not be previously detected (Lee et al., 2020). The possible metabolic mechanisms of these compounds must be elucidated in subsequent studies.

### Polyphenol Metabolism of Soybean Roots

Polyphenols are biosynthesized in living plants to facilitate different activities, such as forming a defense system against exogenous matters, nutrient accumulation, and microorganism interaction in the rhizosphere of the plant (Sugiyama et al., 2017). Malonyl isoflavones, which are produced when symbiotic organisms in the rhizosphere secrete isoflavones in the form of aglycones or glycosides, account for most soybean polyphenols (Sugiyama et al., 2017). The metabolic responses to the synthesis of polyphenols in soybean plants can be altered by endogenous and exogenous factors, such as leaf maturation at the late-growth stage and defense pressure due to biological infections in the rhizosphere (Mun et al., 2021; Ling et al., 2022). Accordingly, other polyphenols can be generated from existing isoflavones *via* different endogenous enzymatic pathways (Ha et al., 2019). CMS and its derivatives have been converted from daidzein and its derivatives (daidzin and malonyl daidzin) *via* the internal cyclization of the molecule (Uchida et al., 2017). The corresponding enzymes were encoded employing transgenic plants, and the plant genes were expressed by heterologous microorganisms (Sohn et al., 2021). CMS occurrences have been largely elucidated, although the occurrences of CMSN have rarely been verified *via* mass spectrometry and NMR (Le-Van, 1984; Yuk et al., 2011b). Thus far, the presence of M-CMSN or its isomers has not been reported. The metabolic flux for generating M-CMSN in the ARs of soybean was higher than that in normal roots (Figure 4). We confirmed the presence of the isomer of M-CMSN *via* NMR analysis, and this represents a unique phenomenon in soybean ARs owing to the chemical induction for converting isoflavones into CMS derivatives. Many different polyphenols, including isoflavones, flavones, flavonols, and coumestan, as well as their corresponding derivatives, have been identified in soybean leaves (Song et al., 2014), while the less-diverse polyphenols have been detected in soybean roots. Similar to the composition of the isoflavones in soybean leaves, malonyl daidzin accounted for the highest proportion (~60%) in soybean roots. Many flavonols and glycosides are present in the leaves, but no flavonol was detected in the soybean ARs and the roots of the soybean planted in AS (data not shown). The isoflavone content of soybean leaves largely increased during the cultivation period of up to 120 days (Rha et al., 2021) probably because of the changes in the biosynthetic flux in mature soybean plants (Dhaubhadel et al., 2003). CMS was detected after 90 and 120 days in field-grown soybean leaves, and this is a well-known phenomenon that is due to the endogenous tolerance of a mature soybean plant to stress (Tripathi et al., 2016; Yun et al., 2016). However, no specific biosynthetic pathways have been revealed for generating M-CMSN and its isomers; thus, further studies are required to elucidate the unconventional occurrences of these compounds in soybean AR cultures.

Leguminous plants interact with different microorganisms by exchanging metabolites and signals to maintain their metabolic system. Alternatively, infectious biotic and abiotic stresses can be modulated in the rhizosphere, following the secretion of the aforementioned modified polyphenols, to facilitate the viability of the plant. The newly isolated compounds, which can influence biological and environmental factors, have been utilized in the pharmaceutical and healthcare industry. Therefore, our approach, AR cultivation, could represent a breakthrough for producing substantial amounts of M-CMSN with pharmaceutical and industrial applications. We attempted to elucidate the biological efficacy of M-CMSN, such as its proliferation effect on human follicle dermal papilla cells (data not shown). However, further concrete evidence would be reported in the future.

## Conclusion

Soybean AR cultivation under abiotic stress induction generated two CMS derivatives, one of which is yet to be identified. AR cultivation can be an effective method for producing leguminous phytochemicals in a timely, cost-effective manner. Leguminous roots preserve their viabilities in distinctive rhizosphere environments *via* root cell–microorganism interactions. Accordingly, different approaches will further verify these findings for the production of significant phytochemicals under biotic or abiotic augmentation. Furthermore, understanding the changes in the metabolic flux in an environment would present substantial clues for a biotechnological approach to plant research in the future.

## Data Availability Statement

The datasets presented in this study can be found in online repositories. The names of the repository/repositories and accession number(s) can be found in the article/Supplementary Material.

## Author Contributions

EJL: conceptualization, data curation, formal analysis, investigation, methodology, resources, validation, visualization, and writing—review and editing. MCS: formal analysis, methodology, resources, and writing—review and editing. C-SR: conceptualization, data curation, formal analysis, software, supervision, validation, visualization, writing—original draft, and writing—review and editing. All authors contributed to the article and approved the submitted version.

## Conflict of Interest

The authors declare the following financial interests/personal relationships which may be considered as potential competing interests: EJL and C-SR declare employment with AMOREPACIFIC Corporation. The remaining author declares that the research was conducted in the absence of any commercial or financial relationships that could be construed as a potential conflict of interest.

## Publisher's Note

All claims expressed in this article are solely those of the authors and do not necessarily represent those of their affiliated organizations, or those of the publisher, the editors and the reviewers. Any product that may be evaluated in this article, or claim that may be made by its manufacturer, is not guaranteed or endorsed by the publisher.

## Abbreviations

1D: one-dimensional
2D: two-dimensional
AR: adventitious root
AS: artificial soil
CE: collision energy
CMS: coumestrol
CMSN: coumestrol monoglucoside/coumestrin
COSY: correlation spectroscopy
DMSO: dimethyl sulfoxide
DP: declustering potential
FA: formic acid
HMBC: heteronuclear multiple bond correlation
HPLC: high-performance liquid chromatography
HSQC: heteronuclear single-quantum coherence
IBA: indole-3-butyric acid
MJ: methyl jasmonate
MS: mass spectrometer
MSO: Murashige and Skoog medium
NMR: nuclear magnetic resonance
PDA: photodiode array
PPFD: photosynthetic photon flux density
PVDF: polyvinylidene fluoride
Q-TOF-HR ESI/MS: quadrupole time-of-flight high-resolution electrospray ionization mass spectrometry
UV: ultraviolet.
