# Supplementary material for: Mass Biosynthesis of Coumestrol Derivatives and Their Isomers via Soybean Adventitious Root Cultivation in Bioreactors
Source: Front Plant Sci. 2022 Jun 21;13:923163. doi: 10.3389/fpls.2022.923163 (PMC9253684; doi:10.3389/fpls.2022.923163)
Supplement: Supplementary file 1 [file Data_Sheet_1.DOCX]

Supplementary Material

## Supplementary Figures


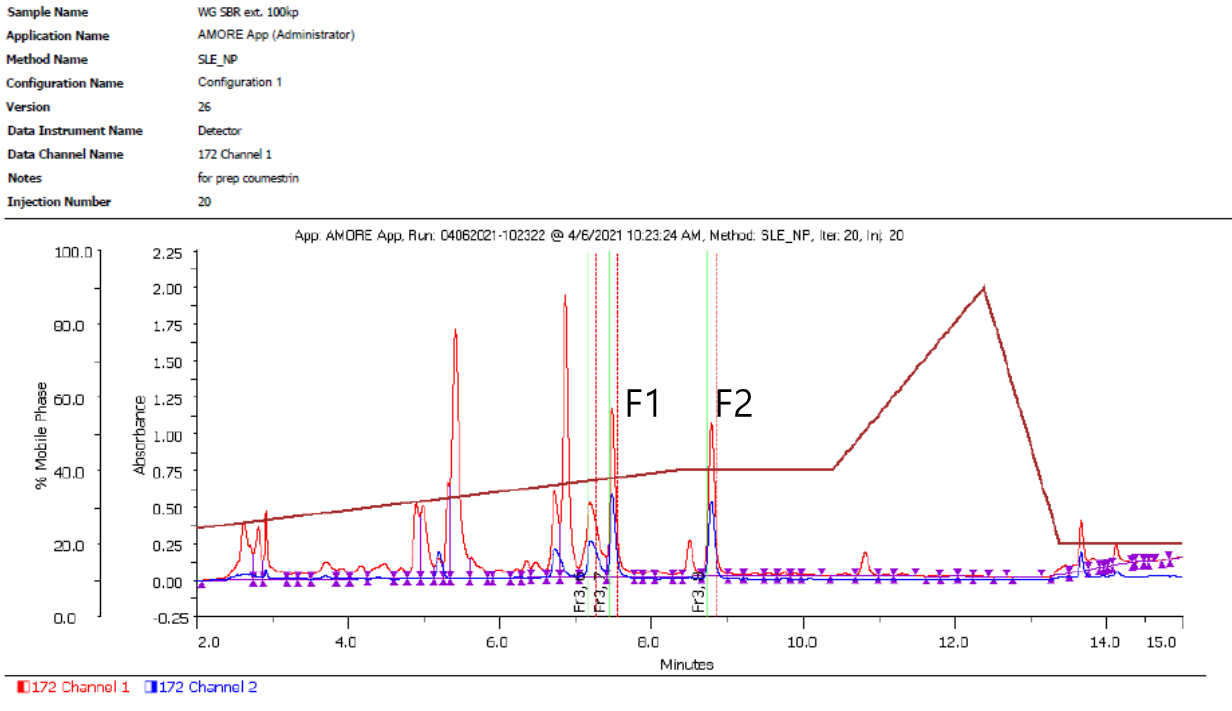


**Supplementary Figure** **S1**. Preparative chromatograms of the coumestrol (CMS) derivatives

**Supplementary Figure** **S2**. Dry masses of soybean and the seedlings
The lowercase letters on the bar indicate significant difference using comparisons for all pair by Tukey-Kramer’s honestly significant difference test at an 𝛼 = 0.05 level using JMP 16 (SAS Institute Inc.)


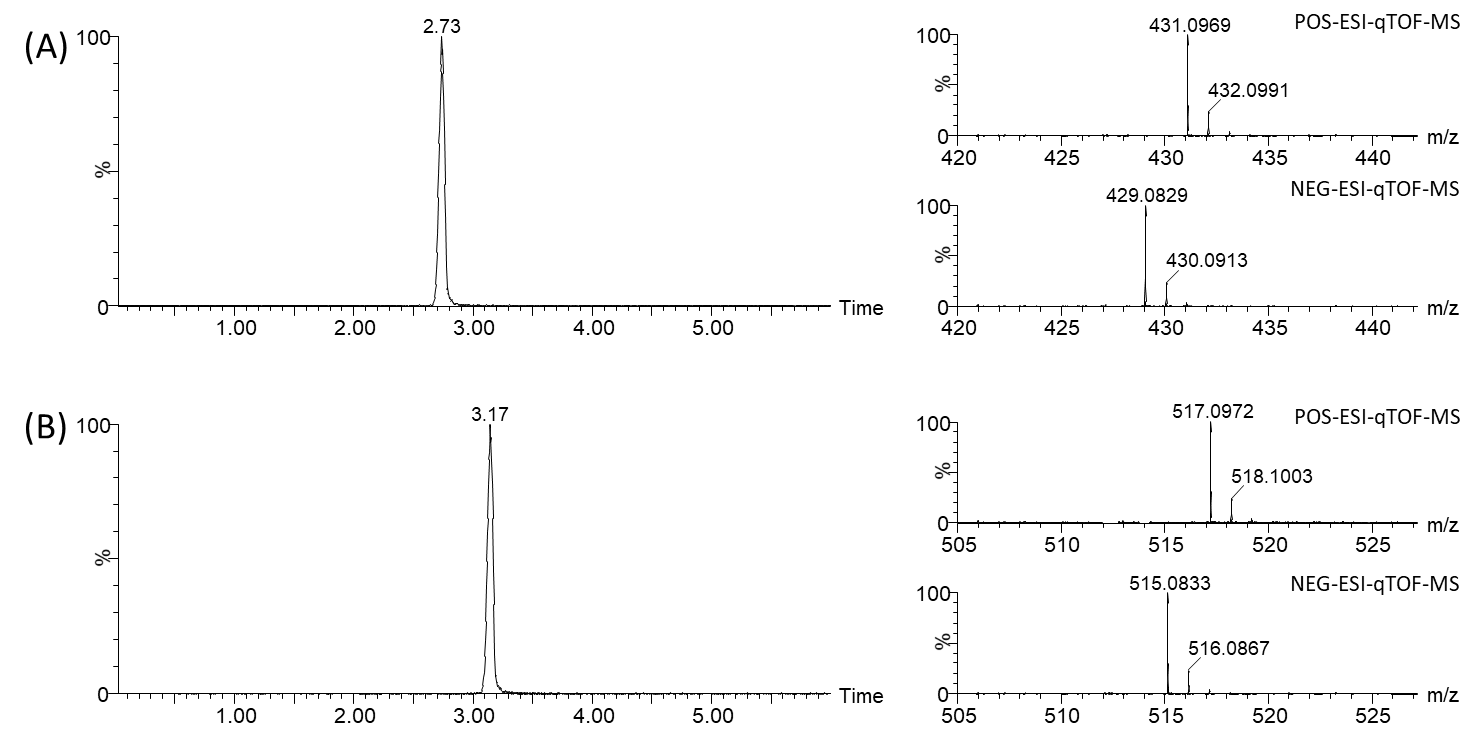


**Supplementary Figure** **S3**. Mass identification of two compounds from the fractions
(**A**) UPLC-Q-TOF-HR ESI/MS chromatogram, positive mass (POS-ESI-qTOF-MS) spectrum, and negative mass (NEG-ESI-qTOF-MS) spectrum, respectively, of coumestrin (1) and coumestrol 9-Glc (2), (**B**) UPLC-Q-TOF-HR ESI/MS chromatogram, POS-ESI-qTOF-MS spectrum, and NEG-ESI-qTOF-MS spectrum, respectively, coumestrin 6′-malonate (3) and coumestrol 9-Glc-6′-malonate (4).


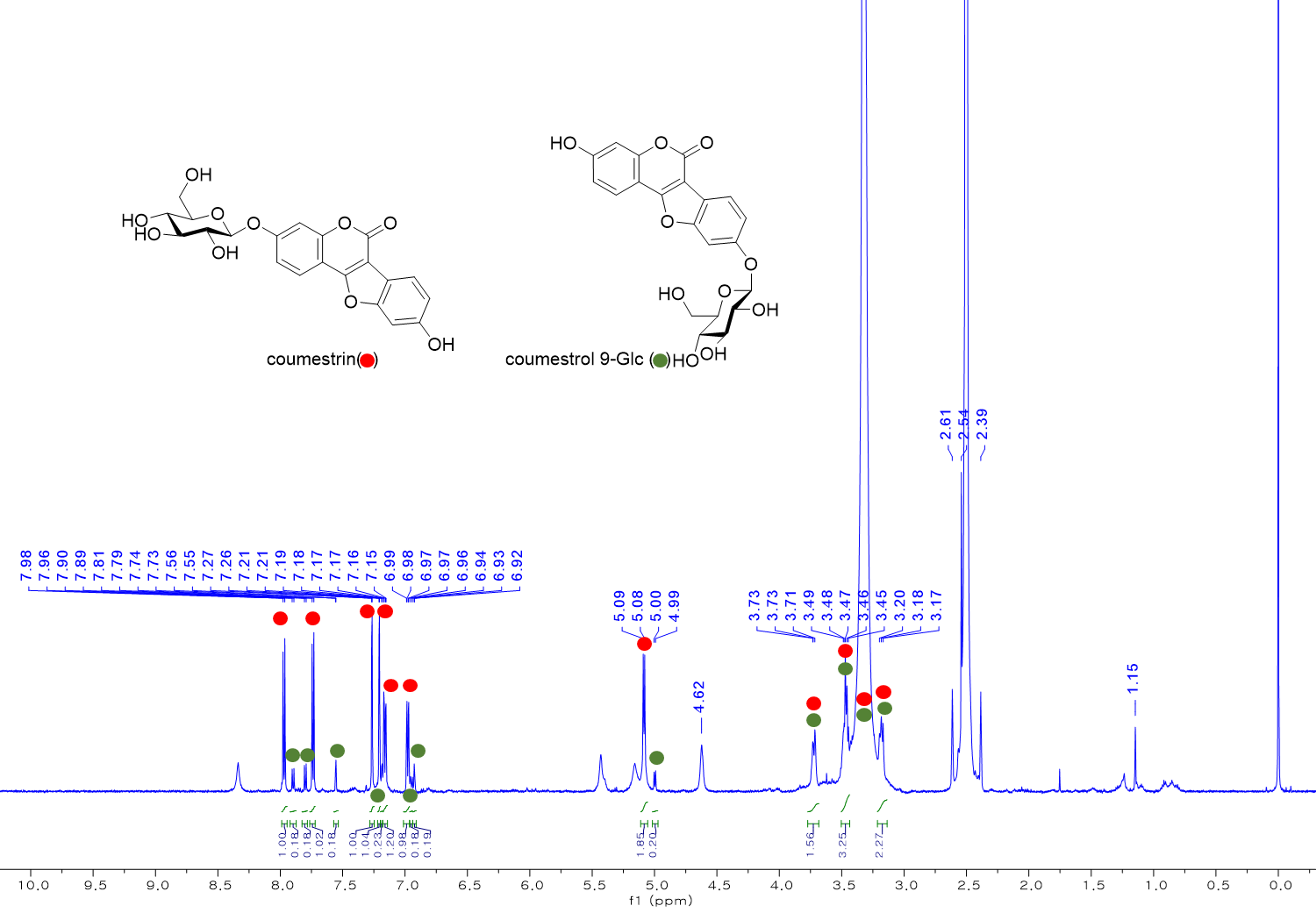


**Supplementary Figure** **S4**. ^1^H-NMR spectrum (600 MHz, DMSO-*d*_6_) of F1


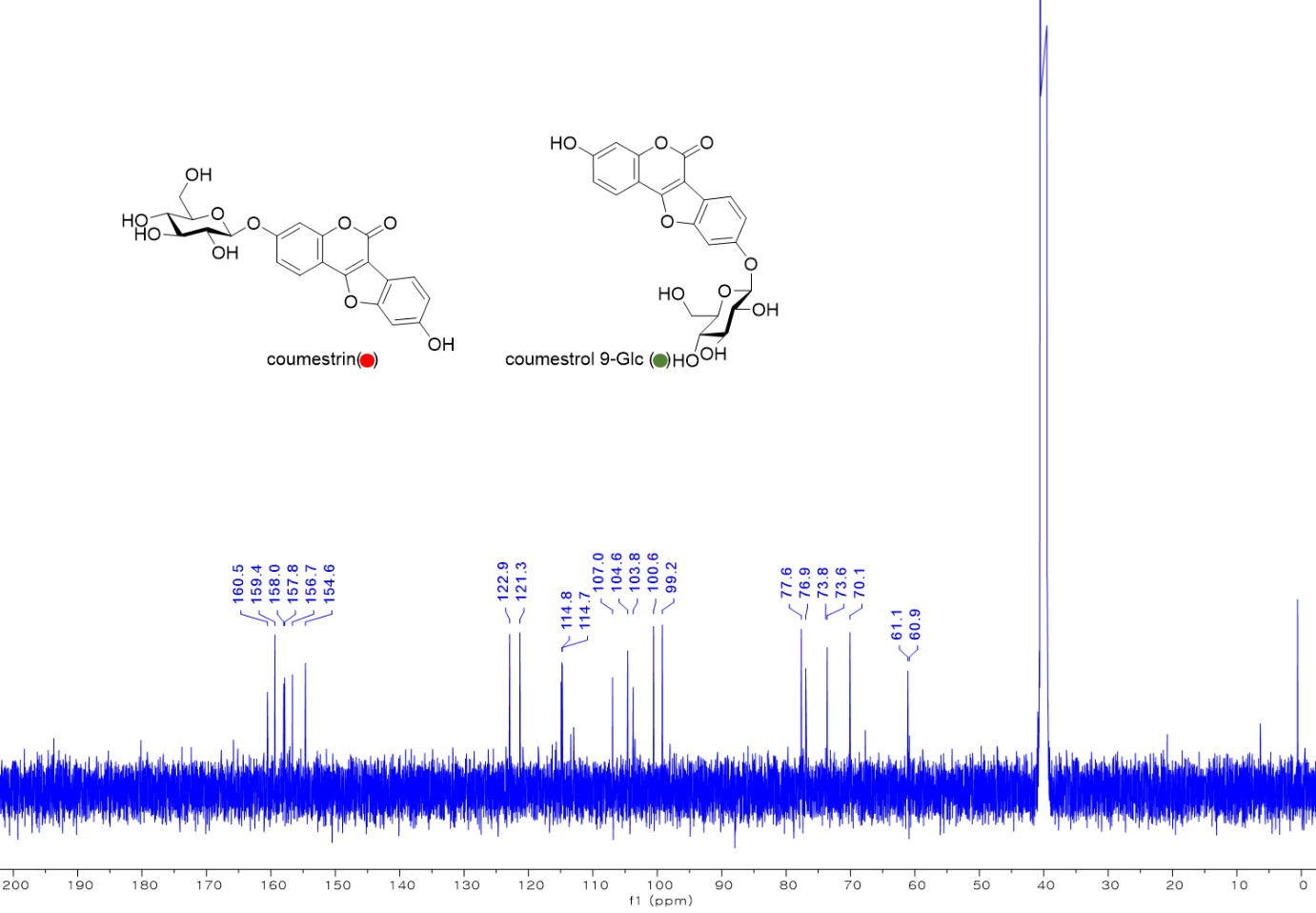


**Supplementary Figure** **S5**. ^13^C-NMR spectrum (150 MHz, DMSO-*d*_6_) of F1


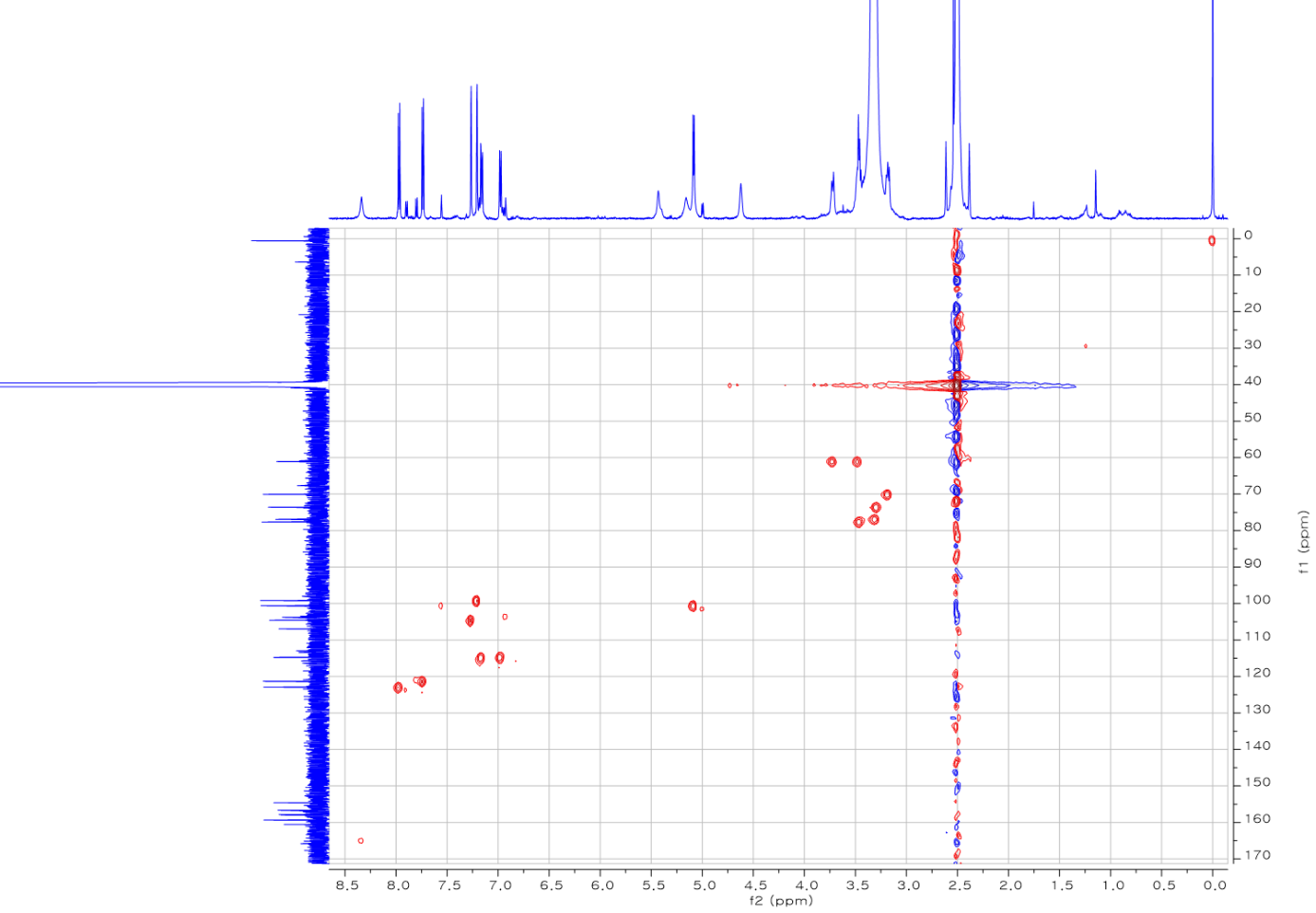


**Supplementary Figure** **S6**. ^1^H-^13^C HMQC spectrum of F1


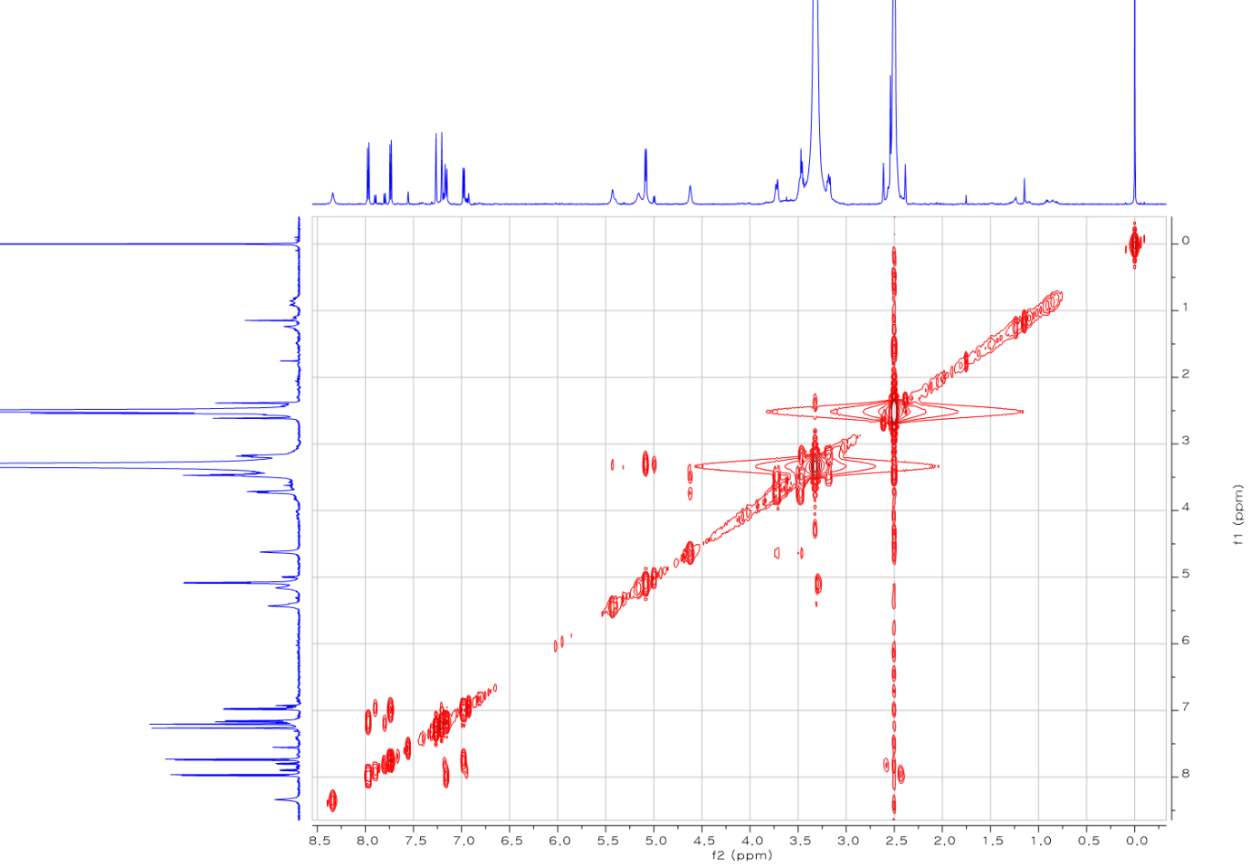


**Supplementary Figure** **S7**. ^1^H-^1^H COSY spectrum of F1


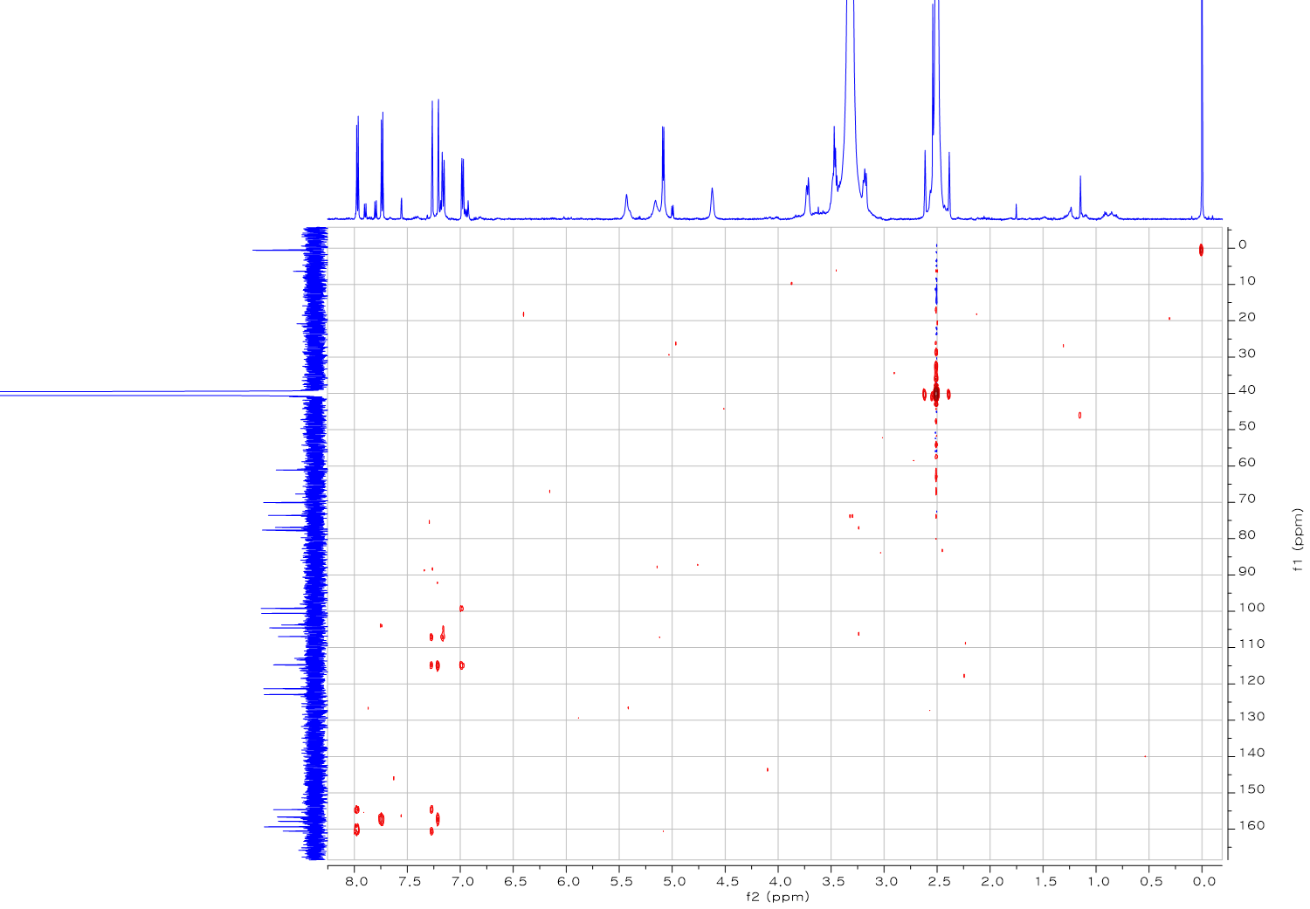


**Supplementary Figure** **S8**. ^1^H-^13^C HMBC spectrum of F1


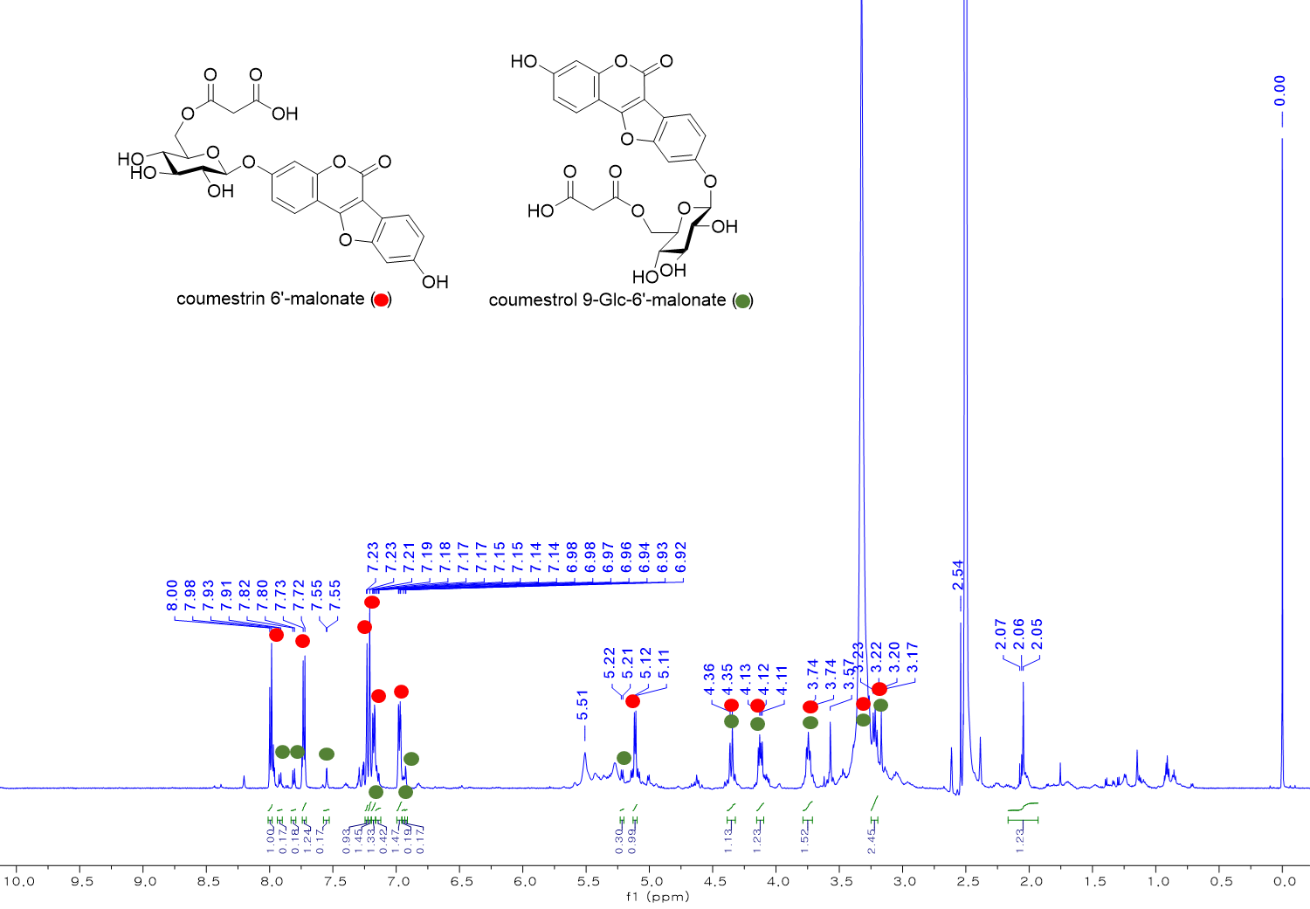


**Supplementary Figure** **S9**. ^1^H-NMR spectrum (600 MHz, DMSO-*d*_6_) of F2


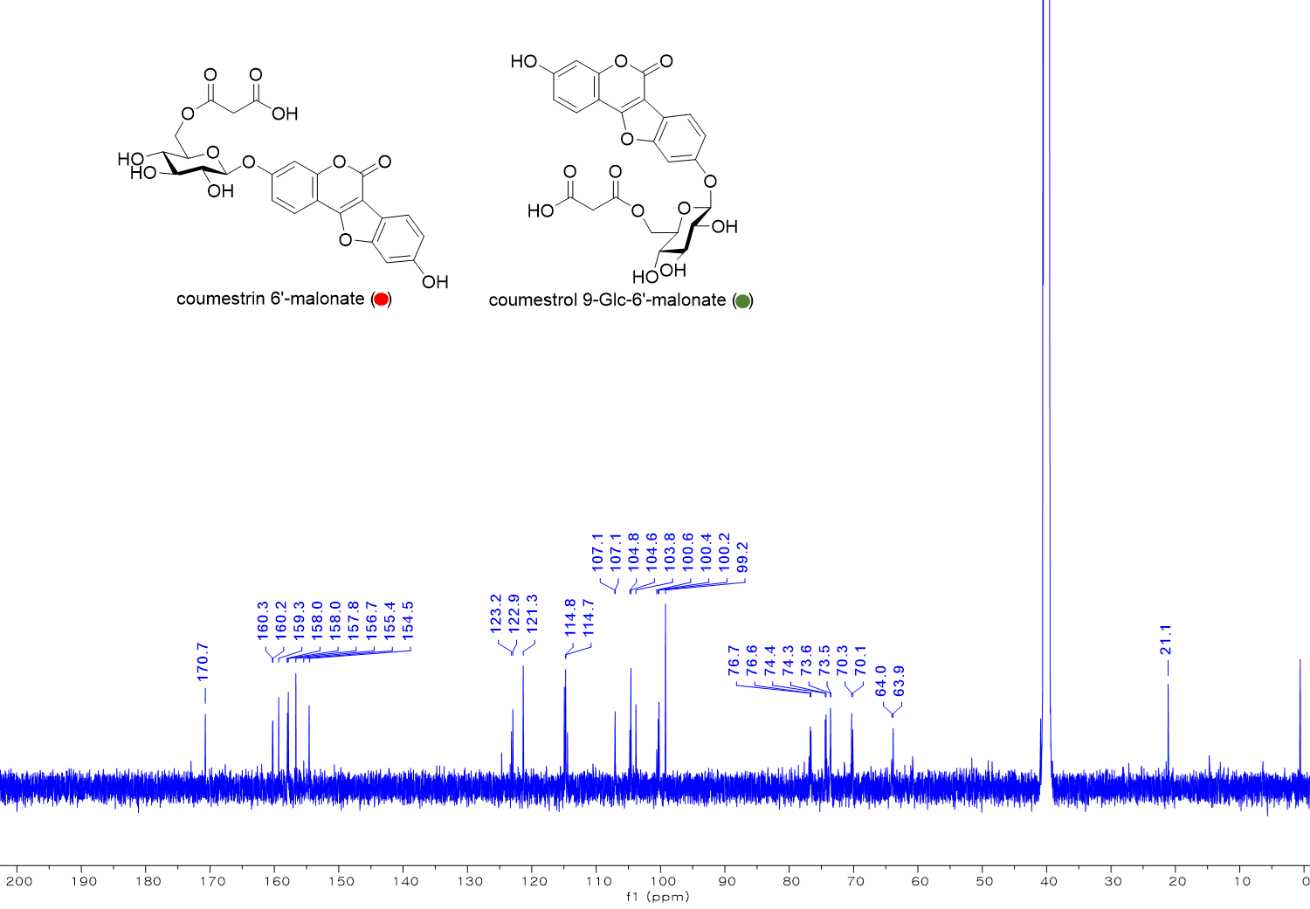


**Supplementary Figure** **S10**. ^13^C-NMR spectrum (150 MHz, DMSO-*d*_6_) of F2


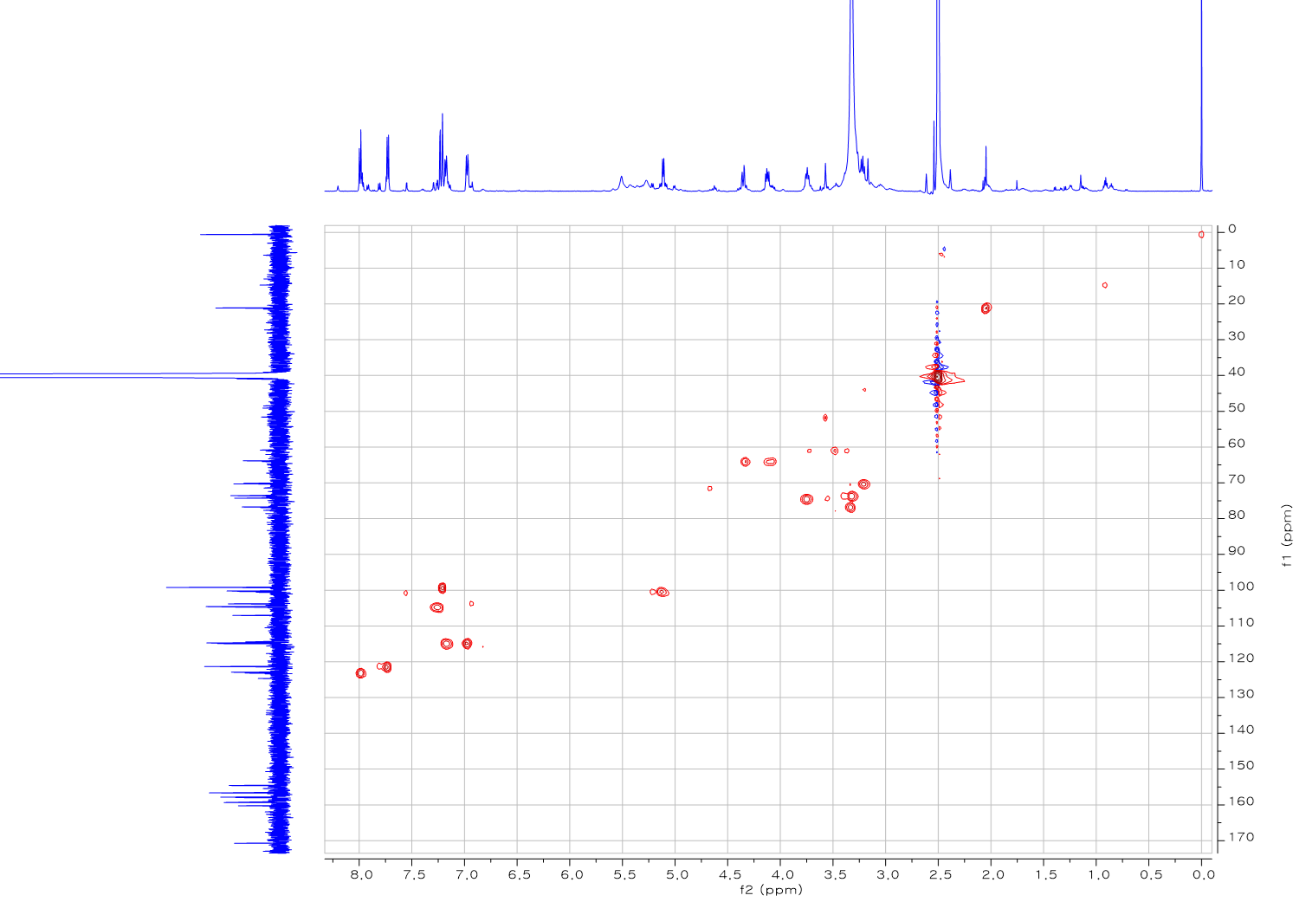


**Supplementary Figure** **S11**. ^1^H-^13^C HMQC spectrum of F2


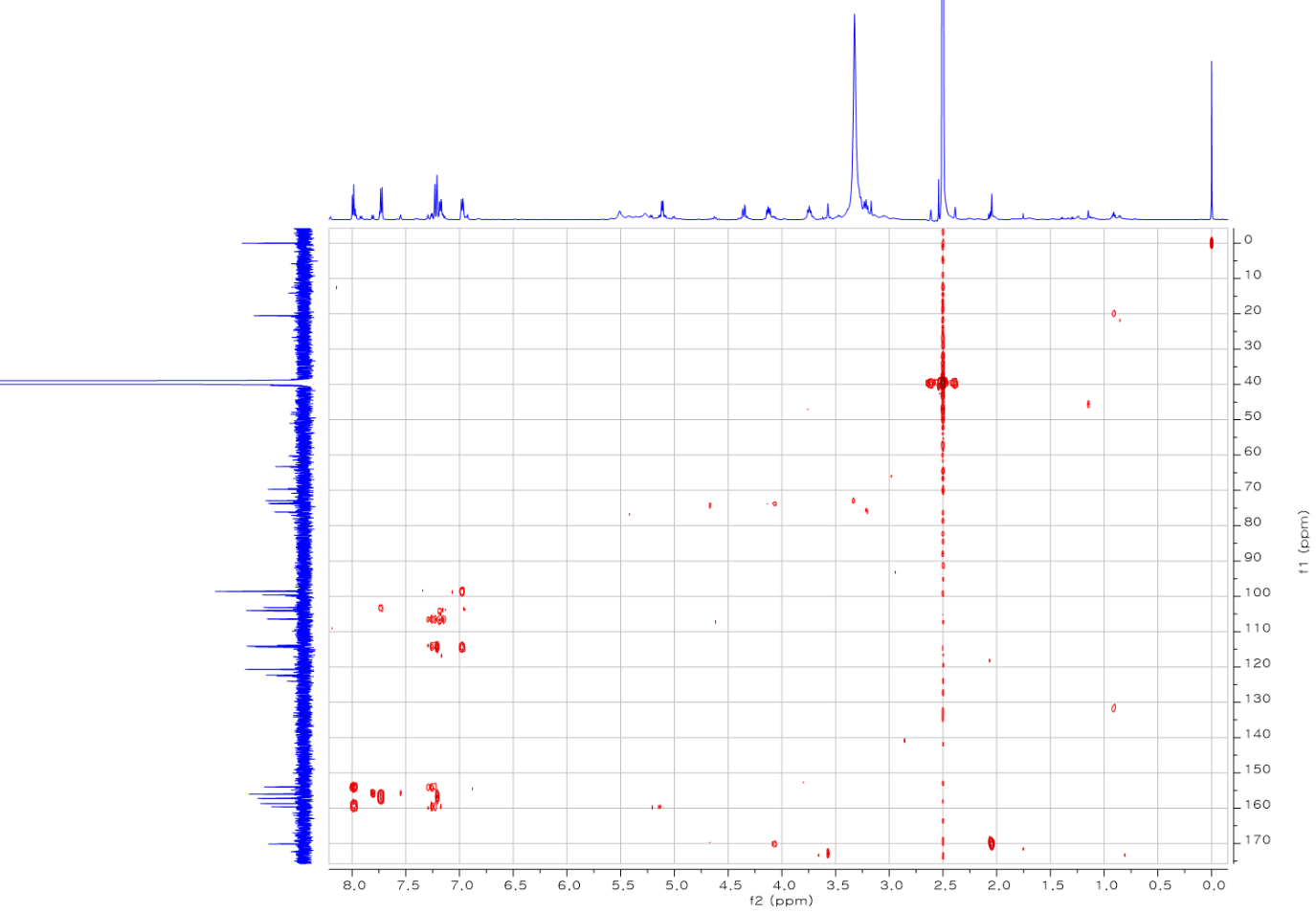


**Supplementary Figure** **S12**. ^1^H-^13^C HMBC spectrum of F2


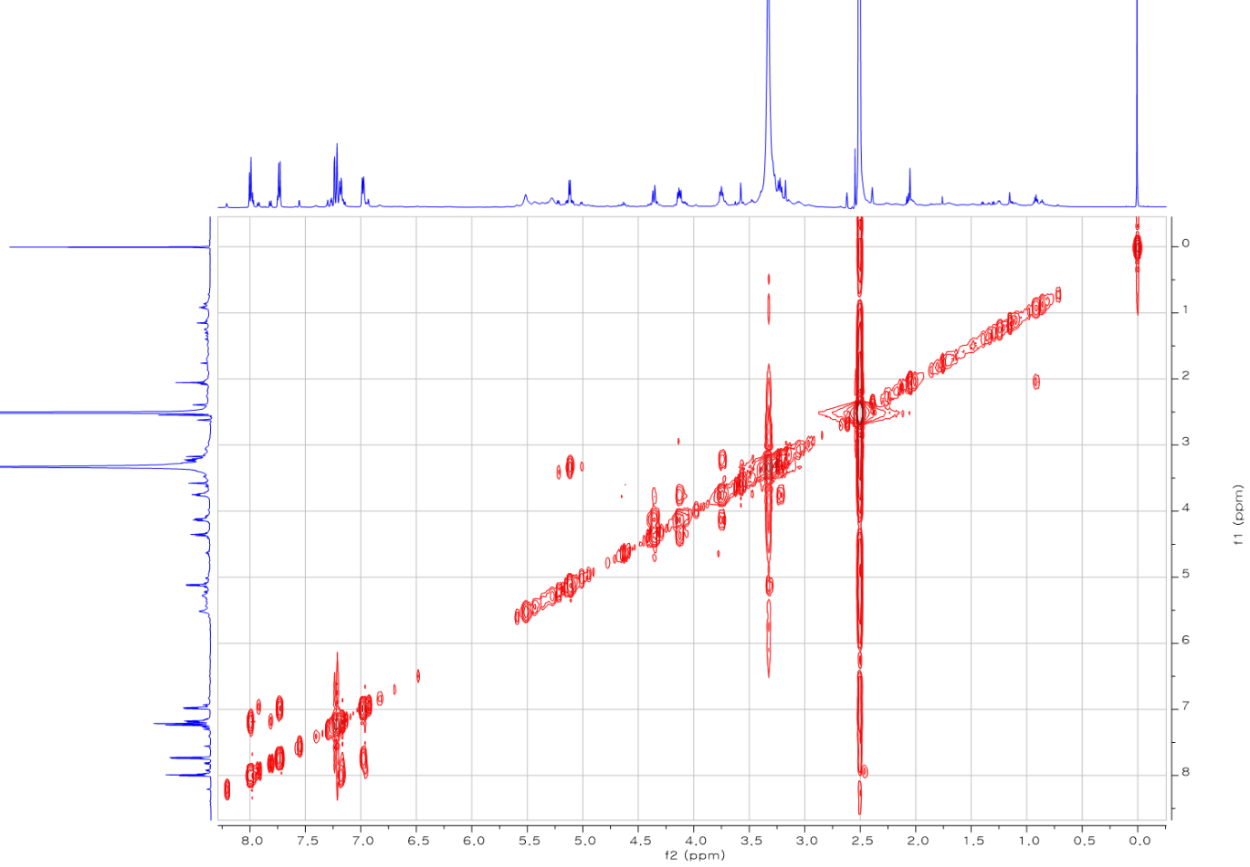


**Supplementary Figure** **S13**. ^1^H-^1^H COSY spectrum of F2

**Supplementary Figure** **S14**. Purity and UV spectra of purified coumestrin (CMSN, F1)
(**A**) Maximum UV absorbance (220–460 nm) of F1. Numbers on the peaks indicate retention time and the following portion (%) of area under curve. (**B**) UV spectra of the corresponding peaks of (A). (**C**) Spectral purity of the peak 5 (retention time, 13.158 min)

**Supplementary Figure** **S15**. Purity and UV spectra of purified malonyl CMSN (F2)
(**A**) Maximum UV absorbance (220–460 nm) of F2. Numbers on the peaks indicate retention time and the following portion (%) of area under curve. (**B**) UV spectra of the corresponding peaks of (A). (**C**) Spectral purity of the peak 5 (retention time, 15.787 min)

**Supplementary Figure** **S16**. Changes in the contents of kaempferol glycosides in the leaves of soybean planted in artificial soil (AS) and the seedling cultures

## Supplementary Movie

**Movie S1**. Quick video of the three-week cultivation period in the 3 L bioreactor. The file is openly available in the repository at https://github.com/chansurha/CMS
